# Treatment patterns and outcomes for second-line therapy in large B-cell lymphoma: results from the LEO CReWE study

**DOI:** 10.1038/s41408-026-01563-2

**Published:** 2026-07-23

**Authors:** Jean L. Koff, Melissa C. Larson, Jonathon B. Cohen, Izidore S. Lossos, Peter Martin, Thomas M. Habermann, Carla Casulo, Samuel Yamshon, Andrea Lo-Rossi, Ashwini Shewade, Connie Lee Batlevi, Anthony Masaquel, Yong Mun, Yucai Wang, Arushi Khurana, Grzegorz S. Nowakowski, Dai Chihara, Eric Mou, Umar Farooq, Dilan Patel, Amy Ayers, Tanner W. Reicks, James R. Cerhan, Christopher R. Flowers, Matthew J. Maurer, Loretta J. Nastoupil

**Affiliations:** 1 Department of Hematology and Medical Oncology, Winship Cancer Institute, Emory University, Atlanta, GA USA; 2 Department of Quantitative Health Sciences, Mayo Clinic, Rochester, MN USA; 3 Division of Hematology, Sylvester Comprehensive Cancer Center, Department of Medicine, University of Miami, Miami, FL USA; 4 Department of Medicine, Weill Cornell Medicine, New York, NY USA; 5 Department of Medicine, New York University Grossman School of Medicine, New York, NY USA; 6 Department of Internal Medicine, Mayo Clinic, Rochester, MN USA; 7 Wilmot Cancer Institute, University of Rochester, Rochester, NY USA; 8 Division of Hematology & Medical Oncology, Weill Cornell Medicine, New York, NY USA; 9 Genentech, Inc, South San Francisco, CA USA; 10 Department of Lymphoma and Myeloma, The University of Texas MD Anderson Cancer Center, Houston, TX USA; 11 Division of Hematology, Oncology, and Bone & Marrow Transplantation, Department of Internal Medicine, University of Iowa, Iowa City, IA USA; 12 Division of Oncology, Washington University School of Medicine, St. Louis, MO USA; 13 Division of Cancer Medicine, The University of Texas MD Anderson Cancer Center, Houston, TX USA; 14 Present Address: CommonSpirit Health, Durango, CO USA

**Keywords:** B-cell lymphoma, Therapeutics

## Abstract

Treatment strategies for relapsed/refractory large B-cell lymphoma (R/R LBCL) have evolved rapidly, yet relationships between therapy selection and outcomes remain poorly defined. The LEO CReWE study evaluated disease characteristics, patterns of care, and outcomes in 1504 patients with R/R LBCL from 2002 to 2022. Of these, 64% received chemoimmunotherapy at second-line (2L). Autologous stem cell transplant (ASCT) and/or chimeric antigen receptor T-cell therapy (CAR-T) were planned at 2L for 984 patients (65%), with 471 (31%) ultimately receiving ASCT and 94 (6%) receiving CAR-T. Complete response (CR) rates at 2L ranged from 24% for lenalidomide-based regimens to 49% for CAR-T; salvage chemotherapy achieved a 44% CR rate overall and 60% among patients who eventually underwent ASCT. Median event-free and overall survival from 2L were 4.3 months and 18.3 months, respectively; survival improved in eras during which CAR-T was available. Patients intended for but unable to receive ASCT or CAR-T experienced poor outcomes comparable to those never intended for curative therapy at 2L. The LEO CReWE study represents the first large-scale depiction of 2L treatment patterns and outcomes for LBCL in the modern era, inclusive of *all* treatment approaches, and underscores the ongoing need for more effective therapies despite the advent of CAR-T.

## Introduction

Large B-cell lymphomas (LBCLs) comprise a group of molecularly and clinically heterogeneous tumors that have recently undergone major shifts in classification and treatment paradigm. The 5th edition of the World Health Organization Classification of Haematolymphoid Tumors defines 18 distinct LBCL entities, the most common of which is diffuse LBCL, not otherwise specified (DLBCL, NOS) [1]. With increased understanding of molecular features associated with distinct pathobiology and clinical outcomes, classification systems have evolved to incorporate factors such as cell-of-origin and chromosomal translocations of *MYC* and *BCL2* (so-called “double-hit” lymphomas) into categorization of LBCL subtypes. Most patients with newly diagnosed LBCL receive an anthracycline-based chemotherapy regimen with rituximab, but outcomes remain disparate. While ~60% of patients are cured with frontline chemoimmunotherapy, the remainder either fail to respond to frontline treatment or subsequently relapse.

Historically, patients with relapsed and/or refractory (R/R) LBCL have experienced poor outcomes and limited therapeutic options. Prior to the recent approvals of novel, targeted therapies for LBCL, most patients with R/R disease were treated with salvage chemoimmunotherapy (CIT) in the second line, followed by high-dose therapy with autologous stem cell transplant (ASCT) for fit patients with good response [2]. The overall response rate with this approach is approximately 60%, with fewer than half of patients alive at 3 years [3]. Outcomes have been found to be especially poor for those patients with primary refractory disease, relapse within 12 months of a frontline regimen, or with insufficient response to salvage chemotherapy, with median overall survival (OS) of just 6 months [3–5].

Treatment options for R/R LBCL have transformed dramatically in recent years, particularly with the advent of CD19-targeted chimeric antigen receptor T cell therapy (CAR-T). CAR-T cells were initially approved as third-line-or-later treatment (3L + ) in 2017, and subsequent randomized studies of LBCL patients with early first relapse demonstrated superior efficacy of axicabtagene ciloleucel and lisocabtagene maraleucel over a standard-of-care approach, including platinum-based salvage CIT followed by consolidative ASCT for responding patients [6–10]. These trials led to CAR-T approval in early 2022 as second-line therapy (2L) in patients who have R/R disease within 12 months of frontline treatment or are not candidates for ASCT. Despite encouraging response rates and improvement in OS in R/R LBCL patients, however, CAR-T remains underutilized: significant resources and time are required to manufacture an autologous product, and cellular therapies must be administered in specialized centers, thus limiting access to certain communities and populations [11, 12].

Recent approvals for several novel targeted agents alone or in combination regimens have further multiplied therapeutic options for patients with R/R LBCL. These include polatuzumab vedotin, approved in combination with bendamustine and rituximab (BR) in 2019 in 3L+ for LBCL [13]; tafasitamab, approved in combination with lenalidomide in 2020 following studies in 2L and 3L in LBCL patients who were not candidates for intensive therapy [14]; loncastuximab tesirine, approved in 2021 for R/R LBCL in the 3L+ setting [15]; and the bispecific antibodies epcoritamab and glofitamab, approved in 2023 for 3L+ treatment of LBCL [16, 17]. Given the rapid development of new therapies in this space, there is a paucity of large-scale efforts that incorporate the full heterogeneity of disease features, patient-level factors, and therapeutic approaches to characterize R/R LBCL patient outcomes within the contemporary treatment landscape.

To address this knowledge gap, we developed the Lymphoma Epidemiology of Outcomes Consortium for Real World Evidence (LEO CReWE) cohort of patients with R/R LBCL to investigate disease characteristics, patterns of care, and survival outcomes in patients receiving two or more lines of systemic therapy across modern treatment eras. In this study we sought to answer the following questions: (1) What characterizes patients with R/R LBCL across treatment eras? (2) What are contemporary response rates and survival outcomes of patients receiving 2L therapies? (3) How has the availability of new therapies impacted patient outcomes over time? (4) What benchmarks for future clinical trials can be derived from this large, U.S.-based patient cohort? (5) Which populations have the greatest unmet need in R/R LBCL?

## Subjects and Methods

### Study design and patient selection

We developed a multicenter cohort of patients with R/R LBCL managed by expert clinical practice across eight academic centers in the U.S. participating in the LEO Cohort Study (NCT02736357; https://leocohort.org/), which was IRB-approved at all sites. LEO centers include Cornell University, Emory University, Mayo Clinic Rochester, MD Anderson, University of Iowa, University of Miami, University of Rochester Medical Center, and Washington University. The current study is an observational study based on secondary use of data collected in the LEO Cohort or from retrospective LEO Consortium institutional databases (see Supplementary Fig. 1). All patient management for those not treated on a clinical trial protocol, including treatment choices, follow-up, use of re-biopsy and imaging, were per practice of the treating physicians.

Eligible patients for this analysis were at least 18 years old and received second-line therapy (2L+ ) between 2002 and 2022, regardless of timing of progression/relapse or treatment received. Patients with post-transplant lymphoproliferative disorder, histologic transformation, primary central nervous system lymphoma, primary mediastinal B-cell lymphoma, Burkitt lymphoma, or plasmablastic lymphoma were excluded. Demographics, prognostic factors, therapy details (including intent for ASCT and/or CAR-T), and outcomes, including treatment response and survival, were abstracted for all lines of therapy (see *Supplementary Materials* for more detail). Each treatment line was assigned to a broader treatment category, as detailed in Supplementary Table 1. Receipt of consolidative ASCT was not considered a treatment category per se but instead was treated as a separate exposure variable; conversely, receipt of CAR-T was categorized as “immune effector cell therapy” regardless of whether or not preceding therapy for disease control was received within the same treatment line. Treatment eras were defined as pre-CAR-T (2002–2010), CAR-T available via clinical trial (2011–2017), and post-FDA approval of CAR-T (2018–2022).

### Statistical analysis

The primary endpoints of interest were event-free survival (EFS) and overall survival (OS). EFS was defined as the time from initiation of 2L until disease progression, relapse, initiation of a new line of therapy due to lack of efficacy, or death from any cause. OS was defined as the time from initiation of 2L to death from any cause. Secondary endpoint definitions are described in *Supplementary Materials*. Responses were assessed via Lugano or Cheson criteria by positron emission tomography (PET) scan, computed tomography (CT) scan, and/or by clinician assessment. Time-to-event variables were estimated using the Kaplan-Meier method. Differences between subgroups were evaluated using an unadjusted Cox proportional hazards models. All models and results are presented with 95% confidence intervals (CI). *P*-values are not reported as there were no formal hypotheses tested.

## Results

### Cohort characteristics

Eligible patients were initially diagnosed between January 3, 2002, and June 21, 2022. Of 1736 patients initiating 2 L for R/R disease, 1504 patients met inclusion criteria as shown in the flow diagram (Supplementary Fig. 2). Patient characteristics at 1L systemic therapy are shown in Supplementary Table 2. Median age at initiation of 2L was 62 years, and 65% were male; 11% were non-White, and 8% were Hispanic. High-grade B-cell lymphoma (HGBL) histology was present in 15% of all cases. IPI was available for 1151 (77%) patients at time of 2L therapy, with 52% having IPI 3–5. Median time from diagnosis to 2L was 8.7 months, with 823 (55%) patients not having achieved complete response (CR) to 1L, 282 (19%) patients relapsing <12 months after 1L, and 299 (27%) patients relapsing ≥12 months after 1L. Full details at 2L are in Table 1 and Supplementary Table 3, and stratified by treatment era in Supplementary Table 4; full details at 3L are in Supplementary Table 5.

**Table 1:** Patient characteristics at second-line therapy (2 L) for large B-cell lymphoma (LBCL).

	Overall (*N* = 1504)
***Demographics***	
**Age at diagnosis (years)**	
Mean (SD)	60.6 (13.0)
Median (IQR)	62 (53, 70)
Range	18–91
18–59	549 (36.5%)
60–69	480 (31.9%)
70–79	380 (25.3%)
80+	95 (6.3%)
**Gender**	
Female	531 (35.3%)
Male	973 (64.7%)
**Race**	
White	1280 (85.1%)
Black or African American	92 (6.1%)
Asian	43 (2.9%)
American Indian or Alaskan Native	2 (0.1%)
Other	33 (2.2%)
Unknown/Missing	54 (3.6%)
**Ethnicity**	
Non-Hispanic	1220 (81.1%)
Hispanic or Latinx	114 (7.6%)
Unknown/Missing	170 (11.3%)
***Disease Characteristics***	
**Stage**	
I or II	274 (19.9%)
III or IV	1104 (80.1%)
Missing data	126
**ECOG performance status**	
0–1	1067 (83.4%)
2–4	213 (16.6%)
Missing data	224
**Serum lactate dehydrogenase**	
Not elevated	537 (45.5%)
Elevated	643 (54.5%)
Missing data	324
**Extranodal sites**	
0–1	1014 (70.1%)
2+	433 (29.9%)
Missing data	57
**IPI score**	
0–1	242 (21.0%)
2	311 (27.0%)
3–5	598 (52.0%)
Missing data	353
**Subtype**^a^	
DLBCL	1218 (81.0%)
HGBL	93 (6.2%)
Double- or triple-hit	193 (12.8%)
**Cell of origin by Hans algorithm**	
GCB	656 (53.7%)
Non-GCB	565 (46.3%)
Missing data	283
**FISH rearrangement**	
No double-hit	858 (84.3%)
Double-hit: rearrangements of MYC and BCL2	114 (11.2%)
Triple-hit: rearrangements of MYC, BCL2 and BCL6	46 (4.5%)
Missing data	486
**Relapsed/refractory status**	
Achieved CR to 1L, relapsed <12 months	282 (18.8%)
Achieved CR to 1L, relapsed ≥12 months	399 (26.5%)
Did not achieve CR to 1L	823 (54.7%)
***Treatment Characteristics***	
**Prior anthracycline status**	
Yes	1436 (95.5%)
No	68 (4.5%)
**Maximum treatment lines abstracted**	
Range	2–18
**Treatment era**	
2002–2010	275 (18.3%)
2011–2017	571 (38.0%)
2018–2022	658 (43.8%)
**Time from diagnosis to 2L (months)**	
Mean (SD)	17.4 (24.0)
Median (IQR)	8.8 (5.6, 18.2)
Range	0.131 - 228.8
**Treatment at 2L**	
Salvage chemotherapy	962 (64.0%)
CNS-directed regimen	149 (9.9%)
Immune effector cell therapy	95 (6.3%)
Novel therapy	94 (6.2%)
Frontline-like regimen	84 (5.6%)
Minimal therapy	51 (3.4%)
ImID-containing regimen without chemotherapy	49 (3.3%)
Polatuzumab vedotin +/- CD20 +/- bendamustine	20 (1.3%)
**Stem cell transplant (SCT) at 2L**	
Autologous	471 (31.3%)
Allogeneic	21 (1.4%)
None	1012 (67.3%)
**Autologous SCT at any treatment line**	
Yes	581 (38.6%)
No	923 (61.4%)
**CAR-T at any treatment line**	
Yes	394 (26.2%)
*Prior autologous SCT*	7 (0.5%)
No	1110 (73.8%)
**Clinical trial**	
Treated off clinical trial	1280 (86.0%)
Treated on clinical trial	208 (14.0%)
Missing data	16

1L first-line, *CAR-T* chimeric antigen receptor T-cell therapy, *CNS* central nervous system, *CR* complete response, *DLBCL* diffuse large B-cell lymphoma, *ECOG* Eastern Cooperative Oncology Group, *FISH* fluorescent in situ hybridization, *GCB* germinal center B cell-like, *HGBL* high-grade B-cell lymphoma, *ImID* immunomodulatory drug, *IPI* International Prognostic Index, *IQR* interquartile range, *SD* standard deviation

^a^Best available subtype based on WHO classification at the time of diagnosis, augmented by FISH testing (clinical and research) where available.

### Treatment patterns for second-line therapy

**Fig. 1:**
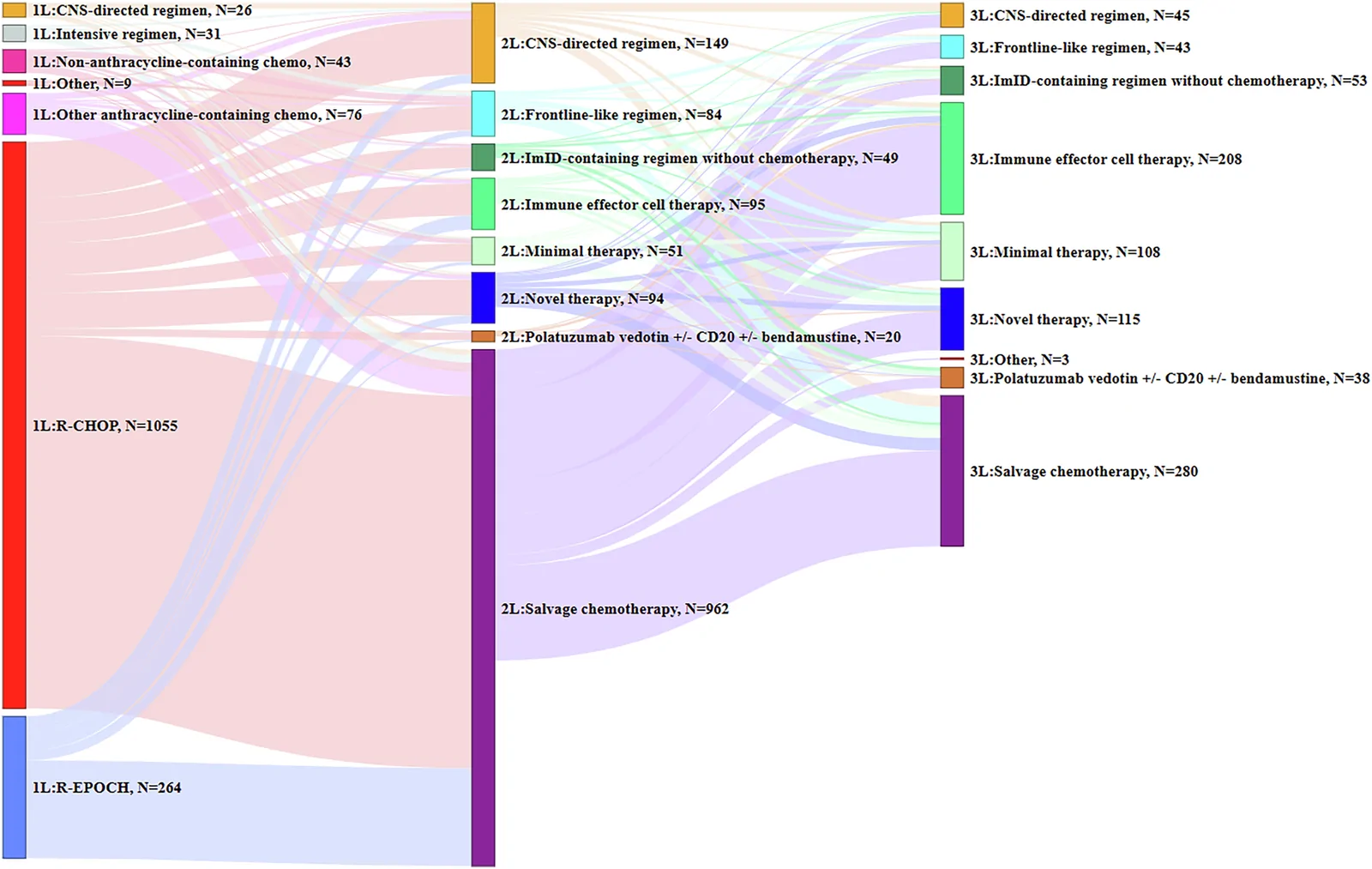
Sankey plot of treatment patterns across lines of therapy. 1L first-line, 2L second-line, 3L third-line, CNS central nervous system, ImID immunomodulatory drug (lenalidomide for all except *n* = 3 cases using pomalidomide), R-CHOP rituximab, cyclophosphamide, doxorubicin, vincristine, and prednisone, R-EPOCH rituximab, cyclophosphamide, doxorubicin, vincristine, and prednisone.

Treatment patterns in 1L showed a predominance of anthracycline-based regimens (70% R-CHOP, 18% R-EPOCH), reflecting the standard of care for LBCL. As shown in Fig. 1, 2L regimens were more variable, but most patients who received anthracycline-based regimens in 1L went on to receive salvage chemotherapy with or without anti-CD20 therapy in 2L. Further details on treatment sequencing can be explored via an interactive Sankey plot application at link.

**Fig. 2:**
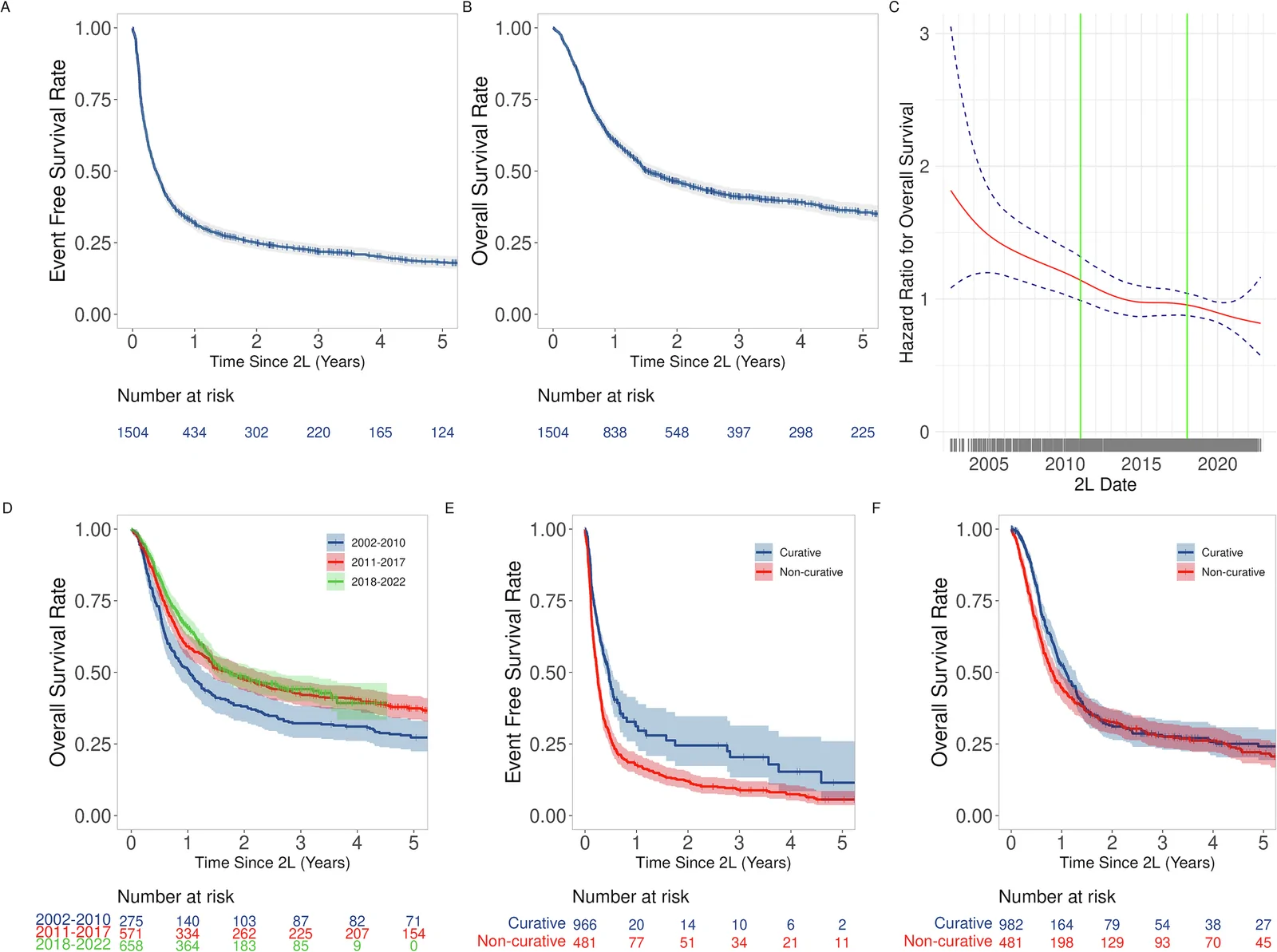
Survival curves for LBCL patients treated with second-line therapy (2L) 2002-2022. Event-free survival (EFS) (**A**) and overall survival (OS) (**B**) in entire cohort. **C** Hazard ratio for overall survival over time, adjusted for age and sex. **D** OS by treatment era. EFS (**E**) and OS (**F**) for patients who did not ultimately receive curative therapy (i.e., CAR-T or stem cell transplant) in 2L, whether curative therapy was intended but not received (blue), or never intended (red).

Treatment type at 2L is shown in Table 1. Salvage chemotherapy with or without anti-CD20 therapy was the most common (64%), with a breakdown of 2L salvage regimens shown in Supplementary Table 3. Other therapy types included CNS-directed regimens (9.9%), immune effector cell therapy (6.3%), off-label therapies with or without chemotherapy (6.2%), frontline-like regimens (e.g., R-CHOP, 5.6%), immunomodulatory (IMiD)-containing regimens without chemotherapy (3.3%), and polatuzumab vedotin with or without bendamustine and/or anti-CD20 (1.3%). Across all therapy types, 2L was received at an academic medical center in 84% cases (75% cases at a LEO center), and 208 patients (14%) received 2L on a clinical trial.

### Characteristics of patients intended for curative therapy

ASCT and/or CAR-T was planned at 2L for 984 patients (65%), of whom 471 received ASCT, 94 received CAR-T, and 21 received allogeneic transplant at 2L. A breakdown of reasons for which patients intended for curative therapy in 2L did not ultimately receive it is found in Supplementary Table 6, with inadequate response or progression on salvage therapy being the most common reason. Disease characteristics of patients who received ASCT at 2L were largely similar to those in patients who received CAR-T at 2L (Table 2; characteristics for patients who underwent ASCT or CAR-T at 3L in Supplementary Table 7). However, patients who received CAR-T in 2L had shorter median time from diagnosis, were less likely to achieve CR to 1L (24% vs. 64%), and were more likely to have a high-grade histologic subtype (28% vs. 11%) than patients who underwent ASCT at 2L. As expected, patients who received CAR-T in 2L were more likely to participate in a 2L clinical trial, and uptake of CAR-T in 2L increased over time, with the vast majority of patients receiving CAR-T at 2L between 2018 and 2022. A total of 581 and 394 patients received ASCT and CAR-T, respectively, at any line of treatment.

**Table 2:** Characteristics of LBCL patients who underwent autologous stem cell transplant (autoSCT) or CAR-T at second-line therapy (2L).

	AutoSCT (*N* = 471)	CAR-T (*N* = 94)
**Age (years)**		
18–59	212 (45.0%)	38 (40.4%)
60+	259 (55.0%)	56 (59.6%)
**Stage**		
I or II	83 (19.0%)	23 (27.1%)
III or IV	353 (81.0%)	62 (72.9%)
Missing data	35	9
**ECOG performance status**		
0–1	390 (92.2%)	80 (92.0%)
2–4	33 (7.8%)	7 (8.0%)
Missing data	48	7
**Serum lactate dehydrogenase**		
Elevated	165 (43.3%)	54 (61.4%)
Not elevated	216 (56.7%)	34 (38.6%)
Missing data	90	6
**B symptoms**		
Yes	32 (6.8%)	6 (6.4%)
None reported	439 (93.2%)	88 (93.6%)
**Extranodal sites**		
0–1	334 (73.4%)	61 (65.6%)
2+	121 (26.6%)	32 (34.4%)
Missing data	16	1
**IPI score**		
0–1	93 (26.0%)	21 (25.9%)
2	131 (36.6%)	19 (23.5%)
3–5	134 (37.4%)	41 (50.6%)
Missing data	113	13
**Subtype**^a^		
DLBCL	416 (88.3%)	65 (69.1%)
HGBL	25 (5.3%)	6 (6.4%)
Double- or triple-hit	30 (6.4%)	23 (24.5%)
**Relapsed/refractory status**		
Achieved CR to 1L, relapsed <12 months	103 (21.9%)	11 (11.7%)
Achieved CR to 1L, relapsed ≥12 months	199 (42.3%)	11 (11.7%)
Did not achieve CR to 1L	169 (35.9%)	72 (76.6%)
**Time from diagnosis to 2L (months)**		
Median (IQR)	13 (8–27)	7 (5-12)
**Treatment era**		
2002–2010	96 (20.4%)	0
2011–2017	208 (44.2%)	4 (4.3%)
2018–2022	167 (35.4%)	90 (95.7%)
**2L treatment prior to auto SCT**		
Salvage chemotherapy	387 (82.2%)	-
CNS-directed regimen	54 (11.5%)	-
Novel therapy	19 (4.0%)	-
Frontline-like regimen	6 (1.3%)	-
ImID-containing regimen without chemotherapy	3 (0.6%)	-
Minimal therapy	1 (0.2%)	-
Polatuzumab vedotin +/- CD20 +/- bendamustine	1 (0.2%)	-
**Bridging therapy prior to CAR-T**		
Yes	-	18 (19.1%)
No	-	76 (80.1%)
**CAR-T product**		
Axicabtagene ciloleucel	-	62 (66.0%)
Lisocabtagene maraleucel	-	5 (5.3%)
Tisagenlecleucel	-	9 (9.6%)
Other/Unknown	-	18 (19.1%)
**Pre-infusion response**		
CR (complete response)	266 (57.1%)	8 (8.8%)
PR (partial response)	147 (31.5%)	19 (20.9%)
SD (stable disease)	19 (4.1%)	5 (5.5%)
PD (progressive disease)	12 (2.6%)	40 (44.0%)
Not assessed	18 (3.9%)	15 (16.5%)
Unable to determine	4 (0.9%)	4 (4.4%)
Missing data	5	3
**Clinical trial**		
Treated off clinical trial	391 (83.9%)	41 (43.6%)
Treated on clinical trial	75 (16.1%)	53 (56.4%)
Missing data	5	0

*1L* first-line, *CAR-T* chimeric antigen receptor T-cell therapy, *CNS* central nervous system, *DLBCL* diffuse large B-cell lymphoma, *ECOG* Eastern Cooperative Oncology Group, *HGBL* high-grade B-cell lymphoma, *ImID* immunomodulatory drug, *IPI* International Prognostic Index, *IQR* interquartile range

^a^Best available subtype based on WHO at the time of diagnosis, augmented by FISH testing (clinical and research) where available.

### Characteristics of subgroups of interest

Patient characteristics at 2L in subgroups of interest are shown in Table 3. As expected, elderly patients (age ≥80 years) were more likely to have performance status >1 (33% vs. 13–19% in younger age groups) and less likely to receive aggressive 2L regimens such as CNS-directed therapy, salvage CIT, and CAR-T; they were more likely to receive IMiD-containing regimens (21% vs 0–5%) and polatuzumab-based regimens (7% vs. 0–2%) than younger age groups. There were no striking differences observed based on cell-of-origin subtype or IPI score, although patients with non-germinal B cell-like (non-GCB) subtype were slightly more likely to receive CNS-directed treatment and slightly less likely to receive salvage regimens at 2L than patients with GCB DLBCL; patients with IPI 0–1 were slightly more likely to receive CAR-T at 2L compared to patients with higher IPI scores. Compared to patients with DLBCL histology, patients with rearrangements of *MYC* and *BCL2* with or without *BCL6* rearrangement (double- or triple-hit lymphoma) were more likely to receive anthracycline-containing regimens (8% vs. 3%) or CAR-T (12% vs. 6%) at 2L, but slightly less likely to receive salvage CIT (60% vs. 65%).

**Table 3:** Patient characteristics at second-line therapy (2L) in subgroups of interest.

	Age 18–59 (*N* = 549)	Age 60–79 (*N* = 860)	Age 80 + (*N* = 95)	GCB (*N* = 332)	Non-GCB (*N* = 310)	Double-/ triple-hit (*N* = 193)	DLBCL (*N* = 1218)	HGBL (*N* = 93)	IPI Group: 0–1 (*N* = 242)	IPI Group: 2 (*N* = 311)	IPI Group: 3–5 (*N* = 598)
**Age group**											
18–59	-	-	-	100 (30.1%)	106 (34.2%)	63 (32.6%)	440 (36.1%)	46 (49.5%)	167 (69.0%)	118 (37.9%)	116 (19.4%)
60+	-	-	-	232 (69.9%)	204 (65.8%)	130 (67.4%)	778 (63.9%)	47 (50.5%)	75 (31.0%)	193 (62.1%)	482 (80.6%)
**Stage**											
I or II	99 (19.8%)	162 (20.5%)	13 (15.3%)	71 (22.4%)	54 (18.6%)	33 (19.1%)	228 (20.3%)	13 (15.5%)	152 (64.1%)	54 (17.4%)	7 (1.2%)
III or IV	402 (80.2%)	630 (79.5%)	72 (84.7%)	246 (77.6%)	236 (81.4%)	140 (80.9%)	893 (79.7%)	71 (84.5%)	85 (35.9%)	257 (82.6%)	581 (98.8%)
Missing data	48	68	10	15	20	20	97	9	5	0	10
**IPI score**											
0–1	167 (41.6%)	72 (10.6%)	3 (4.3%)	54 (19.7%)	52 (19.5%)	28 (18.8%)	206 (22.2%)	8 (11.1%)	-	-	-
2	118 (29.4%)	181 (26.6%)	12 (17.4%)	78 (28.5%)	74 (27.7%)	37 (24.8%)	250 (26.9%)	24 (33.3%)	-	-	-
3–5	116 (28.9%)	428 (62.8%)	54 (78.3%)	142 (51.8%)	141 (52.8%)	84 (56.4%)	474 (51.0%)	40 (55.6%)	-	-	-
Missing data	148	179	26	58	43	44	288	21	-	-	-
**Treatment at 2L**											
Salvage chemotherapy	393 (71.6%)	539 (62.7%)	30 (31.6%)	229 (69.0%)	202 (65.2%)	115 (59.6%)	790 (64.9%)	57 (61.3%)	69 (69.8%)	205 (65.9%)	354 (59.2%)
CNS-directed	57 (10.4%)	88 (10.2%)	4 (4.2%)	23 (6.9%)	31 (10.0%)	14 (7.3%)	123 (10.1%)	12 (12.9%)	18 (7.4%)	33 (10.6%)	57 (9.5%)
Immune effector cell therapy	35 (6.4%)	59 (6.9%)	1 (1.1%)	25 (7.5%)	24 (7.7%)	23 (11.9%)	66 (5.4%)	6 (6.5%)	21 (8.7%)	19 (6.1%)	42 (7.0%)
Novel therapy	24 (4.4%)	55 (6.4%)	11 (11.6%)	10 (3.0%)	20 (6.5%)	10 (5.2%)	82 (6.7%)	2 (2.2%)	15 (6.2%)	21 (6.8%)	39 (6.5%)
Frontline-like regimens	2 (4.0%)	51 (5.9%)	11 (11.6%)	21 (6.3%)	11 (3.5%)	19 (9.8%)	56 (4.6%)	9 (9.7%)	6 (2.5%)	15 (4.8%)	44 (7.4%)
Minimal therapy	15 (2.7%)	26 (3.0%)	10 (10.5%)	8 (2.4%)	5 (1.6%)	5 (2.6%)	41 (3.4%)	5 (5.4%)	9 (3.7%)	9 (2.9%)	12 (2.0%)
ImID-containing regimen without chemotherapy	1 (0.2%)	28 (3.3%)	20 (21.1%)	10 (3.0%)	13 (4.2%)	4 (2.1%)	43 (3.5%)	2 (2.2%)	4 (1.7%)	6 (1.9%)	36 (6.0%)
Polatuzumab vedotin +/- CD20 +/- bendamustine	2 (0.4%)	11 (1.3%)	7 (7.4%)	4 (1.2%)	4 (1.3%)	3 (1.6%)	17 (1.4%)	0 (0.0%)	0 (0.0%)	3 (1.0%)	14 (2.3%)
**Stem cell transplant (SCT) at 2L**											
Autologous	199 (36.2%)	270 (31.4%)	2 (2.1%)	117 (35.2%)	117 (37.7%)	30 (15.5%)	416 (34.2%)	25 (26.9%)	93 (38.4%)	131 (42.1%)	134 (22.4%)
Allogeneic	16 (2.9%)	5 (0.6%)	0 (0.0%)	2 (0.6%)	3 (1.0%)	5 (2.6%)	15 (1.2%)	1 (1.1%)	6 (2.5%)	3 (1.0%)	9 (1.5%)
None	334 (60.8%)	585 (68.0%)	93 (97.9%)	213 (64.2%)	190 (61.3%)	158 (81.9%)	787 (64.6%)	67 (72.0%)	143 (59.1%)	177 (56.9%)	455 (76.1%)
**Clinical trial status**											
Treated off clinical trial	467 (86.3%)	728 (85.3%)	85 (90.4%)	269 (82.3%)	261 (85.0%)	162 (84.8%)	1035 (85.9%)	83 (90.2%)	186 (78.5%)	253 (81.9%)	520 (87.5%)
Treated on clinical trial	74 (13.7%)	125 (14.7%)	9 (9.6%)	58 (17.7%)	46 (15.0%)	29 (15.2%)	170 (14.1%)	9 (9.8%)	51 (21.5%)	56 (18.1%)	74 (12.5%)
Missing data	8	7	1	5	3	2	13	1	5	2	4

*CNS* central nervous system, *DLBCL* diffuse large B-cell lymphoma, *GCB* germinal center B cell-like, *HGBL* high-grade B-cell lymphoma, *ImID* immunomodulatory drug, *IPI* International Prognostic Index, *IQR* interquartile range, *NOS* not otherwise specified.

### Outcomes in entire cohort

At a median follow-up of 48.8 months (IQR 43.0–54.2) from 2L, 921 patients (61%) had died. The median number of total lines of therapy received was 3 (range 2–18). Median EFS from 2L was 4.3 months (95% CI, 4.0–5.0) and 2-year EFS was 25% (95% CI, 22–27%) (Fig. 2A). The 5-year OS from 2L was 36% (95% CI, 33–40%) (Fig. 2B), with median OS of 18.4 months. Overall survival improved over time (Fig. 2C), with improved OS in eras for which CAR-T was available (Fig. 2D). Notably, for patients for whom curative therapy with ASCT or CAR-T was intended but not received, OS was similar to that of patients not intended for curative therapy; however, EFS was improved in the group with curative intent, perhaps due to those patients’ eligibility for more aggressive/effective therapies (Fig. 2E, F). The ORR to 2L was 62% in the 1407 patients evaluable for response; complete response (CR) rate was 43%. Median time to next treatment was 7.8 months, and median duration of response in the 868 responders was 11 months. Lymphoma was the most common cause of death during the follow-up period, with 5-year incidence of 55% compared to unrelated (6%) and unknown (4%) causes. Additional outcomes metrics for the entire cohort are shown in Table 4.

**Table 4:** Outcomes at second-line therapy.

	2-year (95% CI)	5-year (95% CI)	Median (95% CI), months
**Primary and secondary outcomes**			
Progression-free survival	25% (23–28)	18% (16–20)	5 (4–5)
Overall survival	46% (44–49)	36% (33–39)	18 (17–22)
Event-free survival	25% (22–27)	17% (15–20)	4 (4–5)
Time to next treatment	34% (31–37)	27% (24–30)	8 (7–9)
Duration of response (in 868 responders)	42% (39–46)	31% (28–35)	11 (9–15)
**Risk of death by cause (cumulative incidence)**			
Lymphoma	47% (45–50)	55% (52–58)	NA
Other causes	3% (3–4)	6% (5–7)	NA
Missing data or unknown	3% (2–4)	4% (3–5)	NA

*CI* confidence interval, *NA* not applicable.

### Outcomes by type of second-line therapy

Outcomes by subgroups of interest are detailed in Table 5 and Supplementary Table 8. ORR by 2L therapy type ranged from 39% for IMiD-based regimens (comparable to minimal therapy) to 79% for CAR-T; patients who eventually received consolidative ASCT had ORR of 95%. CR rates by type of therapy ranged from 24% for IMiD-based regimens to 49% for CAR-T. CR rate for salvage therapy was 44% in the entire cohort and 60% prior to transplant for patients who underwent ASCT. Despite relatively high response rates, median EFS was under 1 year for all therapy types, with 2-year EFS ranging from 12 to 13% for the few patients who received IMiD- and polatuzumab-based regimens to 33–36% for CNS-directed therapies and CAR-T. However, patients able to receive ASCT had a median EFS of 2.5 years. Five-year OS was 14% in the 20 patients treated with polatuzumab-based regimens at 2L; the other therapy types had 5-year OS ranging from 24 to 43%. Across therapy types, patients treated on clinical trials had higher 2-year EFS compared to patients treated off-trial (34% vs. 23%, respectively) as well as improved median OS from 2L (hazard ratio [HR] = 0.81; 95% CI, 0.67–0.98).

**Table 5:** Outcomes at second-line therapy (2L) in subgroups of interest.

	*N* (%)	2-year EFS	5-year OS	ORR (95% CI)	CR rate (95% CI)	2-year TTNT	2-year DOR	2-year PFS
Treatment at 2L								
Salvage chemotherapy	962 (64%)	25% (22–27)	37% (33–40)	62% (59–65)	44% (41–48)	33% (30–37)	42% (38–47)	25% (22–28)
CNS-directed regimen	149 (10%)	36% (29–45)	43% (35–53)	69% (61–77)	53% (45–62)	55% (47–65)	58% (49–70)	37% (30–46)
Immune effector cell therapy	95^a^ (6%)	33% (24–44)	37% (27–52)	79% (69–86)	49% (39–60)	40% (30–53)	40% (29–55)	33% (24–44)
Autologous stem cell transplant^b^	471 (83%)	53% (49–58)	61% (56–66)	95% (93–97)	83% (79–86)	60% (56–65)	54% (50–60)	54% (49–58)
Novel therapy	94 (6%)	20% (13–30)	26% (17–39)	52% (41–62)	28% (19–38)	26% (18–38)	32% (20–53)	20% (13–31)
Frontline-like regimen	84 (6%)	20% (13–31)	29% (20–43)	65% (53–75)	35% (25–47)	26% (17–38)	34% (22–52)	20% (13–31)
Minimal therapy	51 (3%)	10% (4–23)	36% (24–54)	39% (24–56)	22% (11–40)	13% (6–29)	15% (4–55)	12% (6–25)
ImID–containing regimen without chemotherapy	49 (3%)	12% (5–29)	24% (13–45)	39% (26–54)	24% (14–39)	22% (10–49)	25% (8–74)	13% (6–32)
Polatuzumab vedotin +/− CD20 +/− bendamustine	20 (1%)	13% (4–45)	14% (3–73)	47% (24–71)	35% (15–61)	21% (6–67)	50% (19–100)	13% (4–45)
**Clinical trial**								
Treated off clinical trial	1280 (86%)	23% (21–26)	34% (31–37)	61% (58–64)	42% (39–45)	32% (29–35)	40% (36–44)	24% (21–26)
Treated on clinical trial	208 (14%)	34% (28–42)	42% (35–51)	68% (61–74)	48% (41–55)	43% (36–51)	51% (43–61)	34% (28–42)
**Treatment era**								
2002–2010	275 (18%)	22% (17–27)	27% (22–33)	60% (54–66)	37% (31–44)	34% (29–42)	40% (33–49)	22% (18–28)
2011–2017	571 (38%)	28% (25–32)	37% (34–42)	64% (60–68)	46% (42–50)	37% (33–42)	47% (41–52)	29% (25–33)
2018–2022	658 (44%)	23% (19–26)	39% (33–46)	60% (56–64)	42% (38–46)	30% (26–34)	38% (33–44)	23% (20–27)
**Age (years)**								
18–59	549 (37%)	27% (24–31)	40% (36–45)	61% (57–65)	42% (38–46)	36% (32–41)	46% (41–53)	28% (24–32)
60–69	480 (32%)	26% (22–30)	37% (32–42)	64% (60–69)	48% (44–53)	33% (29–38)	43% (37–49)	26% (23–31)
70–79	380 (25%)	22% (18–27)	32% (27–38)	62% (56–67)	41% (36–46)	32% (27–38)	36% (29–44)	22% (18–27)
80+	95 (6%)	11% (6–21)	10% (4–25)	51% (39–62)	25% (17–36)	26% (17–41)	24% (13–47)	12% (7–22)
**Stage**								
I or II	274 (20%)	29% (24–35)	42% (35–49)	62% (56–68)	45% (39–52)	35% (29–42)	52% (45–62)	30% (25–36)
III or IV	1104 (80%)	24% (22–27)	34% (31–38)	62% (59–65)	43% (40–46)	34% (31–37)	40% (36–44)	25% (22–27)
**IPI score**								
0–1	242 (21%)	38% (32–45)	54% (47–61)	65% (59–71)	51% (45–58)	43% (37–50)	59% (51–68)	38% (32–45)
2	311 (27%)	35% (30–41)	47% (41–53)	69% (64–75)	52% (46–58)	43% (37–49)	51% (44–59)	35% (30–41)
3–5	598 (52%)	17% (14–20)	25% (21–30)	56% (52–60)	35% (31–40)	27% (23–32)	30% (25–36)	17% (14–21)
**Subtype**^c^								
Double/triple-hit	160 (11%)	15% (11–23)	24% (16–36)	52% (44–61)	32% (24–40)	24% (17–34)	32% (22–45)	15% (11–23)
DLBCL	1233 (82%)	27% (24–29)	37% (34–41)	64% (61–66)	45% (42–47)	36% (33–39)	43% (39–47)	27% (25–30)
HGBL	111 (7%)	22% (15–31)	31% (23–42)	54% (44–64)	39% (30–49)	31% (23–43)	45% (34–61)	22% (15–31)
**Cell of origin by Hans criteria**								
GCB	656 (54%)	25% (22–29)	35% (31–39)	63% (59–67)	41% (37–45)	33% (29–38)	43% (38–48)	25% (22–29)
Non–GCB	565 (46%)	26% (23–30)	39% (34–44)	63% (59–67)	46% (42–51)	36% (31–40)	44% (38–50)	27% (23–31)
**Relapsed/refractory status**								
Achieved CR to 1L, relapsed <12 months	282 (19%)	23% (19–29)	41% (35–48)	66% (60–72)	48% (42–54)	32% (26–38)	38% (31–46)	24% (19–29)
Achieved CR to 1L, relapsed ≥12 months	399 (27%)	38% (33–44)	47% (41–54)	85% (81–88)	68% (63–73)	49% (43–55)	46% (40–52)	39% (34–44)
Did not achieve CR to 1L	823 (55%)	18% (16–21)	28% (25–32)	49% (45–52)	28% (25–31)	27% (23–30)	41% (36–46)	19% (16–22)
**Time from diagnosis to 2L (months)**								
0–36	1328 (88%)	22% (20–25)	34% (31–37)	59% (56–62)	40% (37–43)	30% (28–33)	40% (36–44)	23% (21–25)
36+	176 (12%)	41% (34–50)	46% (37–56)	83% (76–88)	65% (57–72)	59% (51–68)	52% (44–63)	43% (36–52)

*CAR-T* chimeric antigen receptor T-cell therapy, *CNS* central nervous system, *CR* complete response, *DLBCL* diffuse large B-cell lymphoma, *DOR* duration of response, *ECOG* Eastern Cooperative Oncology Group, *EFS* event-free survival, *GCB* germinal center B cell-like, *HGBL* high-grade B-cell lymphoma, *ImID* immunomodulatory drug, *IPI* International Prognostic Index, *IQR* interquartile range, *NOS* not otherwise specified, *OS* overall survival, *PFS* progression-free survival, *TTNT* time to next treatment.

^a^All patients received CAR-T except *n* = 1 treated with investigational natural killer (NK) cell product.

^b^Outcomes calculated after receipt of autologous stem cell transplant.

^c^Best available subtype based on WHO at the time of diagnosis, augmented by FISH testing (clinical and research) where available.

### Outcomes in subgroups of interest

As shown in Table 5 and Supplementary Table 8, primary refractory status was strongly associated with treatment and survival outcomes: patients whose first relapse occurred ≥12 months after 1L had improved 2-year EFS, ORR, and CR rates compared to patients who failed to achieve CR to 1L or relapsed within 12 months. IPI score 3–5 was predictive of inferior 2-year EFS, 5-year OS, CR rate, TTNT and DOR compared to IPI score 0–1. 2L outcomes did not vary significantly by cell-of-origin subtype, but double-/triple-hit lymphomas had inferior 2-year EFS, ORR, and CR rates after 2L compared to cases with DLBCL, NOS histology.

## Discussion

In the wake of paradigm-shifting treatment modalities for relapsed/refractory LBCL such as CAR-T, it is crucial to evaluate the effectiveness of new therapeutic options in real-world practice settings to better inform clinical management of this heterogeneous disease. To our knowledge, the current study is the first-ever large-scale attempt to describe patterns of care and outcomes in LBCL 2L in the modern era, as well as the association between patients’ clinical characteristics and these outcomes, *inclusive of all treatment approaches*. The LEO CReWE dataset is unique in its ability to capture changing practice patterns over time, including specific documentation of intent for and receipt of cellular therapies. We found that CR rates to 2L were unfavorable overall, only somewhat better when patients were treated with intensive therapy (e.g., planned for CAR-T and/or ASCT, CR rate 44–53%) compared to other regimens (22–35%). However, patients intended for curative therapy but who did not receive it experienced poor survival outcomes similar to those observed in patients never intended for CAR-T or transplant. As might be expected based on findings from the SCHOLAR-1 study [5], primary refractory status was predictive of inferior response rates and survival; high IPI score and rearrangements of *MYC* and *BCL2* were also associated with worse outcomes. In fact, with the exception of patients with IPI 0–1 and/or ≥36 months between diagnosis and 2L, 5-year overall survival and duration of response remained poor for all patient subgroups. Notably, overall survival increased in treatment eras during which CAR-T was available. However, fewer than 40% of patients achieved survival at 5 years even in the more recent eras, highlighting the need to identify treatment approaches that result in long-term survival for more patients with R/R LBCL.

Platinum-based chemoimmunotherapy followed by ASCT consolidation can be curative for LBCL patients who relapse >12 months after frontline therapy and have chemosensitive disease in 2L. In our cohort, 31% patients underwent ASCT in 2L, but another 26% were intended for curative therapy with either ASCT or CAR-T but ultimately did not receive it; inadequate response to salvage therapy or complications that rendered patients ineligible for transplant accounted for at least 17% of the entire LBCL 2L cohort. Among patients who received CIT in 2L, ORR was 62%, with median EFS of just 4.3 months. Our findings are consistent with previous studies demonstrating that many patients with R/R LBCL do not respond to CIT in 2L [3, 5, 9, 18–21]. Analyzing pooled data from two phase 3 trials and two observational cohorts comprising 636 patients with refractory LBCL with up to 14 years of follow-up, the SCHOLAR-1 study reported ORR to next line of therapy of 26% and median OS of 6.3 months [5]. In another study of 195 LBCL patients treated at 19 centers in the U.S. and Canada, the ORR to 2L CIT was 43%, with 48% patients experiencing disease progression [22]. Similar results have been reported in studies conducted outside the U.S., with response rates of 33–40%, median PFS of 2–4 months, and median OS of 10–11 months in patients with R/R DLBCL who largely received CIT in 2L [20, 23, 24]. The longer median OS we observed for salvage CIT in our cohort (18.4 months) may reflect improved selection of candidates for 2L CIT and ASCT, or the ability to salvage patients with CAR-T or other newer therapies in 3L or beyond. Accordingly, patients who received ASCT in 2L were much more likely to have a later relapse compared to patients who received CAR-T in 2L (42.3 vs. 11.7%), likely reflecting selection of curative therapy modality influenced by clinical trial availability and informed by data from prior studies such as SCHOLAR-1.

CD19 CAR-T has emerged as the preferred 2L approach for fit patients with LBCL whose relapse occurs within 12 months of frontline therapy based on results from the ZUMA-7 and TRANSFORM trials showing superior efficacy of axi-cel and liso-cel, respectively, over CIT and ASCT in this setting. With median 47 months of follow-up, ZUMA-7 reported a CR rate of 65% and median EFS of 10.8 months in the 180 patients on the axi-cel arm (of whom 10 did not receive infusion) [10]. TRANSFORM reported a 74% CR rate and median EFS 29.5 months in 92 patients on the liso-cel arm (90 patients infused), with median 34 months of follow-up [25]. Although several studies have analyzed outcomes of LBCL patients treated with CAR-T outside a clinical trial in 3L+ (as incorporated into a recent meta-analysis [26]), the data for real-world cohorts focused on the 2L setting remain limited. Our study included patients who received 2L therapy between 2002 and 2022 and thus captured cases treated just after axi-cel was initially approved for 2L LBCL treatment in April 2022. In our cohort, 94 patients received CAR-T infusion in 2L, with CR rate 49% and median EFS of 6 months after median follow-up of 24 months. These outcomes approximate those observed in randomized 2L clinical trials given that our cohort included patients treated with axi-cel, liso-cel, and tisagenlecleucel, which failed to outperform CIT and ASCT in 2L in the BELINDA trial [19]. At least 9 other patients in our cohort were intended for CAR-T but unable to receive due to manufacturing failure, inadequate response to bridging therapy, or treatment complications; an additional 66 patients were coded as having inadequate response/progression to salvage therapy or CAR-T bridging, but our retrospective study was unable to discriminate distinct intent for ASCT vs. CAR-T in those cases. These results suggest a higher proportion of patients intended for but unable to receive CAR-T than what can be estimated from clinical trials or even in some emerging real-world datasets. For instance, a recent multi-institutional study of real-world outcomes in LBCL patients treated with axi-cel or liso-cel in 2L reported that of 112 patients leukapheresed, only 2 failed to receive CAR-T infusion [27]. However, because our study does not filter on 2L treatment received a priori, it may offer a more complete evaluation of barriers to curative therapy than datasets whose populations are defined by leukapheresis or infusion of CAR-T product. Since access to CAR-T remains challenging for many patients, especially those who receive care in remote areas and community practices, novel therapies that are both effective and accessible are sorely needed in this space. Taken together, our data underscore the need for improved regimens that strike a better balance between disease control and toxicity, thus maximizing patients’ ability to receive curative therapy in 2L.

Our study does have several limitations common to retrospective studies. First, responses to treatment were assessed by clinicians from data obtained via chart review and thus were subject to potential measurement error. In addition, because treatment planning at 2L often involves consideration of both ASCT and CAR-T for a given patient, our data collection was not designed to discriminate between intent for one curative approach versus another. Finally, results from a large dataset derived from eight U.S. academic cancer centers may not be generalizable to patients with R/R LBCL managed in the community setting or in other countries, given expanded access to emerging therapies and possible overrepresentation of patients with refractory disease in our cohort.

A key strength of this study is its use of patient-level data from a large consortium to define real-world clinical outcomes of more than 1500 patients with R/R LBCL in centers with clinical expertise in lymphoma. Granular information on patients’ demographic and clinical characteristics allowed for comprehensive evaluation of the largest cohort of patients with R/R LBCL to date, including a detailed analysis of patients intended for curative treatment approaches in 2L, whether or not ASCT or CAR-T was actually received. Overall, this study’s extensive subgroup analyses incorporating patient factors and treatment patterns provide points of reference for future prospective studies in specific LBCL populations. Thus, the LEO CReWE analysis represents a historical benchmark for future studies of R/R LBCL in the era of immune effector cell therapy. The suboptimal response and survival outcomes demonstrated here indicate ongoing unmet need for effective treatment approaches for relapsed aggressive B-cell lymphoma in the second line and beyond.

## Supplementary information

Supplemental Material

## Data Availability

These data are not publicly available. Data sharing policies and the process to request LEO data that support the findings of this study can be found on the LEO Cohort website (https://leocohort.org/).
