# Supplementary material for: Treatment patterns and outcomes for second-line therapy in large B-cell lymphoma: results from the LEO CReWE study
Source: Blood Cancer J. 2026 Jul 23;16(1):185. doi: 10.1038/s41408-026-01563-2 (PMC13680380; doi:10.1038/s41408-026-01563-2)
Supplement: Supplementary file 1 [file 41408_2026_1563_MOESM1_ESM.pdf]

Supplemental Material

Supplementary Figure 1.

Study Design Schema

Development of LEO-CReWE LBCL database from LEO Cohort and LEO Consortium

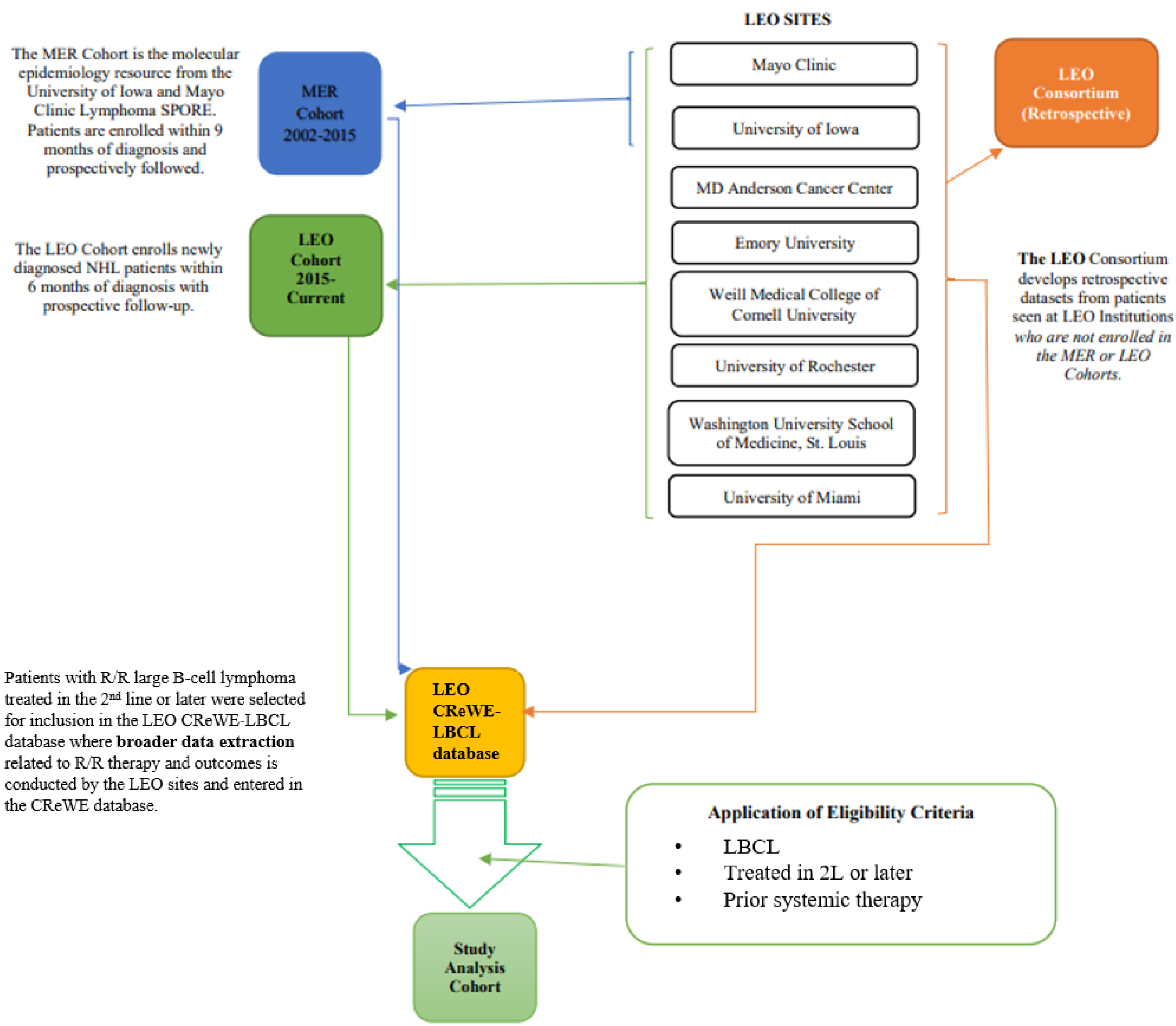

Supplementary Figure 2. Patient Flow Diagram

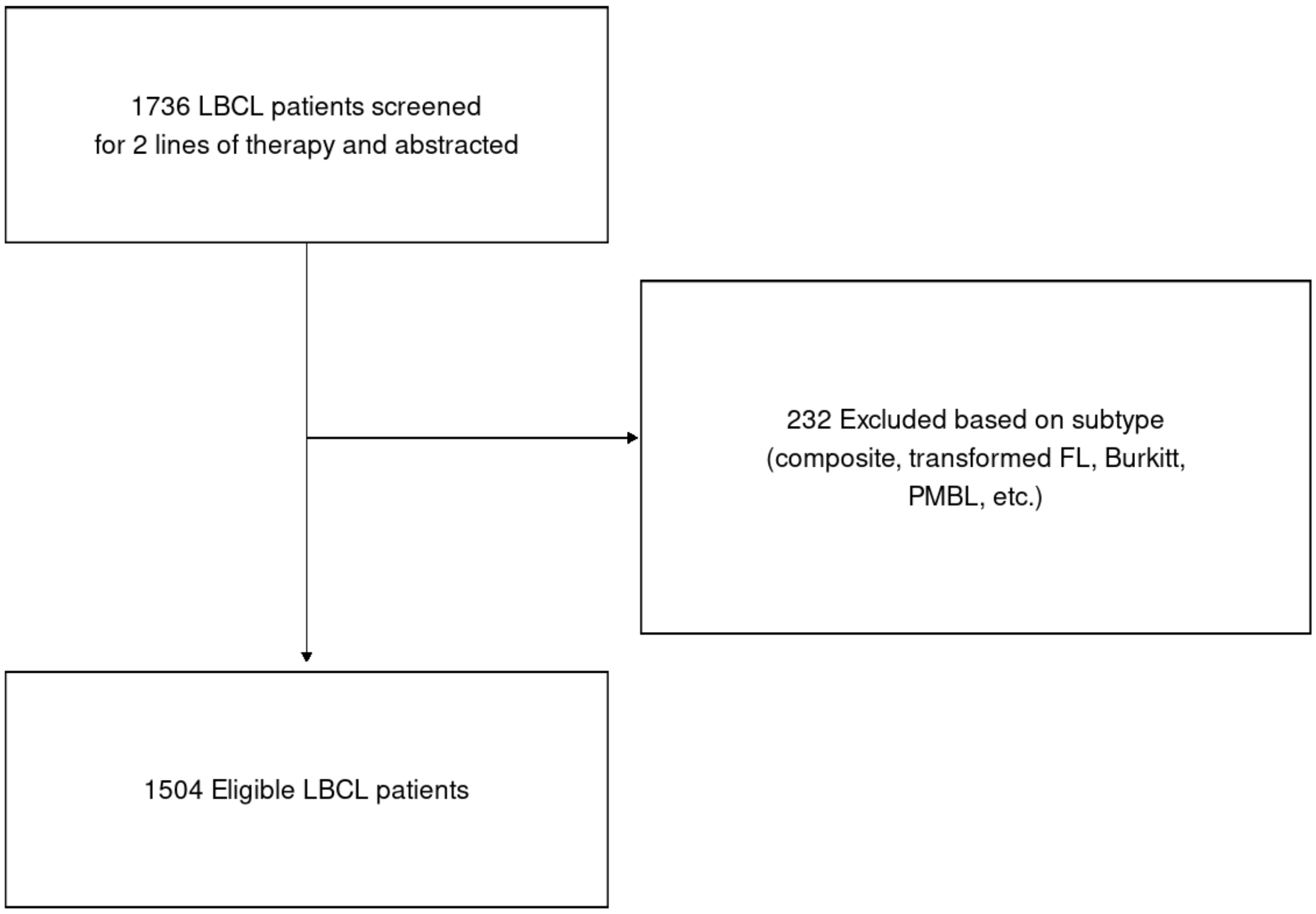

Abbreviations: LBCL, large B-cell lymphoma; FL, follicular lymphoma; PMBL, primary mediastinal B-cell lymphoma

Supplemental Table 1. Treatment categories.

Treatment Category	Agents/Regimens Included
**First-line (1L)**	
R-CHOP	R-CHOP with or without IV or IT methotrexate (MTX)<br>R-mini-CHOP<br>*Modified R-CHOP*<br>R-CHOP + *X* regimens<br>R-CHOP followed by non-CD20 maintenance
R-EPOCH	R-EPOCH without IV MTX
CNS-directed	HD-MTX<br>MATRIX<br>MVP<br>Intrathecal chemotherapy only<br>EPOCH with HD-MTX<br>Other cytarabine + methotrexate regimens
Other anthracycline-containing chemotherapy	Pola-R-CHP<br>*Intensive regimens*<br>HyperCVAD<br>CODOX-IVAC<br>CALGB 10403<br>Vanderbilt<br>*Other non-CHOP, non-EPOCH, anthracycline-containing regimens*
Non-anthracycline-containing chemotherapy	CEOP +/- CD20<br>CVP +/- CD20<br>ESHAP +/- CD20<br>Gem-Ox +/- CD20<br>ICE +/- CD20<br>Bendamustine +/- CD20
Other	*Minimal therapy*<br>CD20 monoclonal antibody monotherapy<br>Surgery
**Second-line and beyond (2L+)**	
Salvage chemotherapy	Bendamustine +/- CD20<br>DHAP +/- CD20<br>ESHAP +/- CD20<br>GDP +/- CD20<br>Gemcitabine-oxaliplatin +/- CD20<br>Gemcitabine-vinorelbine +/- CD20<br>ICE +/- CD20
CNS-directed regimen	HD-MTX<br>MATRix<br>MVP<br>IT chemotherapy only<br>EPOCH or R-ICE with HD-MTX<br>Other cytarabine + methotrexate regimens
Immune effector cell (IEC) therapy	*Any IEC therapy with or without preceding therapies within the same treatment line*<br>CD19 CAR-T<br>Non-CD19 CAR-T<br>Other IEC therapies (e.g., NK cells)
Novel therapy	Loncastuximab tesirine<br>Brentuximab vedotin +/- CD20<br>BTK inhibitors with or without chemotherapy<br>Bispecific antibodies<br>Checkpoint inhibitors<br>ImID in combination with chemotherapy<br>Other small molecule inhibitors with or without chemotherapy<br>Non-polatuzumab, non-brentuximab antibody-drug conjugates<br>Non-rituximab, non-obinutuzumab, non-tafasitamab monoclonal antibodies

Frontline-like regimen	R-CHOP<br>Pola-R-CHP<br>EPOCH +/- CD20<br>Other anthracycline-containing regimens<br>CEOP +/- CD20<br>CEPP +/- CD20<br>CVP +/- CD20<br>*Intensive regimens*<br>HyperCVAD<br>CODOX-IVAC<br>CALGB 10403<br>Vanderbilt
Minimal therapy	Monotherapy with CD20 monoclonal antibody<br>Radiation only<br>Steroids only<br>Surgery
ImID-containing regimen without chemotherapy	Lenalidomide +/- CD20<br>Tafasitamab + lenalidomide<br>Other ImID-based regimens without chemotherapy
Polatuzumab vedotin +/- CD20 +/- bendamustine	Polatuzumab vedotin +/- CD20 monoclonal antibody +/- bendamustine

Abbreviations: CALGB 10403, intensive pediatric regimen from the Cancer and Leukemia Group B 10403 clinical trial; CAR-T, chimeric antigen receptor T-cell therapy; CEOP, cyclophosphamide, etoposide, vincristine, and prednisone; CEPP, cyclophosphamide, etoposide, procarbazine, and prednisone; CHOP, cyclophosphamide, doxorubicin, vincristine, and prednisone; CHP, cyclophosphamide, doxorubicin, and prednisone; CNS, central nervous system; CODOX-M-IVAC, cyclophosphamide, doxorubicin, high-dose methotrexate, ifosfamide, etoposide, and high-dose cytarabine; CVP, cyclophosphamide, vincristine, and prednisone; DHAP, dexamethasone, high-dose cytarabine and cisplatin; EPOCH, etoposide, prednisone, vincristine, cyclophosphamide, and doxorubicin; ESHAP, etoposide, methylprednisolone, high-dose cytarabine and cisplatin; gem-ox, gemcitabine and oxaliplatin; GDP, gemcitabine, dexamethasone and platinum agent; HD-MTX, high-dose methotrexate; hyperCVAD, cyclophosphamide, vincristine, doxorubicin, dexamethasone, methotrexate, and cytarabine; ICE, ifosfamide, carboplatin, and etoposide; ImID, immunomodulatory drug; IT, intrathecal; IV, intravenous; MATRix, methotrexate, cytarabine, thiotepa, and rituximab; MVP, methotrexate, vincristine, and procarbazine; pola, polatuzumab vedotin; R-, rituximab

Supplemental Table 2. Characteristics at diagnosis / 1L treatment.

	Overall (N=1504)
**RUCC 2013 status**	
Missing	47
Metro - Counties in metro areas of 1 million population or more	661 (45.4%)
Metro - Counties in metro areas of 250,000 to 1 million population	183 (12.6%)
Metro - Counties in metro areas of fewer than 250,000 population	215 (14.8%)
Nonmetro - Completely rural or less than 2,500 urban population, adjacent to a metro area	20 (1.4%)
Nonmetro - Completely rural or less than 2,500 urban population, not adjacent to a metro area	28 (1.9%)
Nonmetro - Urban population of 2,500 to 19,999, adjacent to a metro area	133 (9.1%)
Nonmetro - Urban population of 2,500 to 19,999, not adjacent to a metro area	97 (6.7%)
Nonmetro - Urban population of 20,000 or more, adjacent to a metro area	81 (5.6%)
Nonmetro - Urban population of 20,000 or more, not adjacent to a metro area	39 (2.7%)
**Histology**	
ALK-positive large B-cell lymphoma	2 (0.1%)
B cell unclassifiable with features intermediate between DLBCL and Burkitt	13 (0.9%)
DLBCL	1242 (82.6%)
EBV positive DLBCL, NOS	18 (1.2%)
High grade B-cell lymphoma, NOS	53 (3.5%)
High grade, not Burkitt, not classified	20 (1.3%)
High-grade B-cell lymphoma with MYC and BCL2 and/or BCL6 rearrangements	132 (8.8%)
Primary cutaneous DLBCL, leg type	5 (0.3%)
T-cell/histiocyte rich large B-cell lymphoma	19 (1.3%)
**Treatment Group**	
Anthracycline-containing without CD20	2 (0.1%)
CNS-directed	26 (1.7%)
EPOCH +/- CD20	253 (16.8%)
Immune effector cell	1 (0.1%)
Intensive	31 (2.1%)
Minimal therapy	6 (0.4%)
Modified anthracycline-containing 1L with CD20	69 (4.6%)
Non-anthracycline-containing chemo +/- CD20	40 (2.7%)
Novel therapeutic(s) with or without chemotherapy	2 (0.1%)
Other anthracycline-containing regimen with unknown CD20 status	5 (0.3%)
R-EPOCH with IV or IT MTX	11 (0.7%)
R-CHOP	1055 (70.1%)
Salvage chemotherapy +/- CD20	3 (0.2%)
**Detailed Treatment Group**	

1L Trial	55 (3.7%)
Anthracycline-based regimen without CD20	2 (0.1%)
Bendamustine +/- CD20	7 (0.5%)
CEOP +/- CD20	14 (0.9%)
CHOP, unknown CD20 status	13 (0.9%)
CNS-directed	26 (1.7%)
CVP +/- CD20	12 (0.8%)
EPOCH +/- CD20	254 (16.9%)
ESHAP +/- CD20	1 (0.1%)
Gem-Ox +/- CD20	1 (0.1%)
ICE +/- CD20	1 (0.1%)
ImID in combination regimen with chemotherapy	1 (0.1%)
Intensive	31 (2.1%)
Modified RCHOP	9 (0.6%)
No active therapy	1 (0.1%)
Other	1 (0.1%)
Other anthracycline-containing chemotherapy with CD20	4 (0.3%)
Other anthracycline-containing chemotherapy, unknown CD20	5 (0.3%)
Other non-anthracycline-containing chemotherapy/combination	7 (0.5%)
Pola-R-CHP	1 (0.1%)
R monotherapy	2 (0.1%)
R-EPOCH with IT or IV MTX	11 (0.7%)
R-miniCHOP	4 (0.3%)
R-CHOP	959 (63.8%)
R-CHOP with IV or IT CNS-directed therapy	34 (2.3%)
R-CHOP with IV MTX	45 (3.0%)
Surgery	3 (0.2%)

Abbreviations: 1L, first-line treatment; CEOP, cyclophosphamide, etoposide, vincristine, and prednisone; CHP, cyclophosphamide, doxorubicin, and prednisone; CHOP, cyclophosphamide, doxorubicin, vincristine, and prednisone; CNS, central nervous system; CVP, cyclophosphamide, vincristine, and prednisone; DLBCL, diffuse large B-cell lymphoma; EBV, Epstein-Barr virus; ESHAP, etoposide, methylprednisolone, high-dose cytarabine and cisplatin; EPOCH, etoposide, prednisone, vincristine, cyclophosphamide, and doxorubicin; gem-ox, gemcitabine and oxaliplatin; ICE, ifosfamide, carboplatin, and etoposide; IT, intrathecal; IV, intravenous; MER, Mayo Epidemiology Resource; NOS, not otherwise specified; R-, rituximab; WHO, World Health Organization

Supplemental Table 3. Additional data at 2L treatment.

	Overall (N=1504)
**LEO Cohort study site**	
Cornell University	90 (6.0%)
Emory University	114 (7.6%)
University of Iowa	142 (9.4%)
Mayo Clinic	427 (28.4%)
MD Anderson	297 (19.7%)
University of Miami	79 (5.3%)
University of Rochester Medical Center	78 (5.2%)
Washington University	277 (18.4%)
**RUCC 2013 status**	
Metropolitan	1059 (72.7%)
Non-metropolitan	398 (27.3%)
Missing data	47
**B symptoms**	
None Reported	1399 (93.0%)
Yes	105 (7.0%)
Missing or indeterminate	499 (33.2%)
**Bone Marrow Involvement**	
Missing	22
Yes, DLBCL present per path	95 (6.4%)
Yes, indolent/low-grade B-cell only	5 (0.3%)
No (per path confirmation)	466 (31.4%)
Pathology indeterminate	6 (0.4%)
PET/radiographic positive, no biopsy performed	35 (2.4%)
No biopsy/ unable to obtain records	471 (31.8%)
PET/radiographic negative, no biopsy performed	395 (26.7%)
Yes, other	9 (0.6%)
**Bone marrow involvement**	
No	861 (57.2%)
Yes	144 (9.6%)
Missing/Indeterminate	499 (33.2%)
**Histology**	
ALK-positive large B-cell lymphoma	2 (0.1%)
B cell unclassifiable with features intermediate between DLBCL and Burkitt	13 (0.8%)
DLBCL, NOS	1236 (82.2%)
EBV positive DLBCL, NOS	18 (1.2%)
High grade B-cell lymphoma, NOS	70 (4.7%)
High grade, not Burkitt, not classified	18 (1.2%)

High-grade B-cell lymphoma with MYC and BCL2 and/or BCL6 rearrangements	124 (8.2%)
Primary cutaneous DLBCL, leg type	4 (0.3%)
T-cell/histiocyte rich large B-cell lymphoma	19 (1.3%)
**FISH+ for MYC and BCL2 and/or BCL6**	
Missing	493
No	818 (80.9%)
Double- or triple-hit	193 (19.1%)
**FISH+ for MYC and BCL2**	
Missing	486
No	858 (84.3%)
Double-hit	160 (15.7%)
**Double expression of MYC and BCL2**	
No	420 (48.8%)
Double expressor	441 (51.2%)
Missing data	643
**Treatment Group**	
Anthracycline-containing regimen without CD20	1 (0.1%)
Bispecific antibody	2 (0.1%)
CNS-directed	149 (9.9%)
EPOCH +/- CD20	23 (1.5%)
ImID-based regimen without chemotherapy	49 (3.3%)
Immune effector cell	95 (6.3%)
Axicaptagene ciloleucel	62 (4.1%)
Lisocaptagene maraleucel	5 (0.3%)
Tisagenlecleucel	9 (0.6%)
Other / unknown	19 (1.3%)
Intensive	14 (0.9%)
Loncastuximab tesirine	2 (0.1%)
Minimal therapy	51 (3.4%)
Modified anthracycline-containing frontline-like regimen with CD20	2 (0.1%)
Non-anthracycline-containing chemotherapy +/- CD20	168 (11.2%)
Novel therapeutic(s) with or without chemotherapy	90 (6.0%)
Polatuzumab vedotin +/- CD20 +/- bendamustine	20 (1.3%)
R-EPOCH with IV and/or IT MTX	1 (0.1%)
R-CHOP	23 (1.5%)
Salvage chemotherapy +/- CD20	814 (54.1%)
**Detailed Treatment Group**	
Anthracycline-containing chemotherapy, no CD20	1 (0.1%)
Bendamustine +/- CD20	27 (1.8%)
Brentuximab +/- CD20	3 (0.2%)
BTK inhibitor with chemotherapy	4 (0.3%)
BTK inhibitor without chemo	5 (0.3%)

CD19 CAR-T	34 (2.3%)
CD20-CD3 bispecific antibody	2 (0.1%)
CEOP +/- CD20	6 (0.4%)
CEPP +/- CD20	9 (0.6%)
CNS-directed	149 (9.9%)
Checkpoint inhibitor	3 (0.2%)
CVP +/- CD20	5 (0.3%)
DHAP +/- CD20	106 (7.0%)
EPOCH +/- CD20	23 (1.5%)
ESHAP +/- CD20	17 (1.1%)
GDP +/- CD20	64 (4.3%)
Gem-Ox +/- CD20	59 (3.9%)
GN +/- CD20	1 (0.1%)
ICE +/- CD20	511 (34.0%)
ImID in combination with chemo	18 (1.2%)
Intensive	14 (0.9%)
Lenalidomide +/- CD20	24 (1.6%)
Loncastuximab tesirine	2 (0.1%)
Multiple salvage regimens	56 (3.7%)
No active therapy	2 (0.1%)
OTHER	9 (0.6%)
Other monoclonal antibody	4 (0.3%)
Other antibody-drug conjugate	1 (0.1%)
Other anthracycline-containing regimen with CD20	1 (0.1%)
Other cell therapy	8 (0.5%)
Other ImID-based regimen without chemotherapy	1 (0.1%)
Other non-anthracycline-containing chemotherapy/combination	121 (8.0%)
Polatuzumab vedotin +/- CD20 +/- bendamustine	20 (1.3%)
Pola-R-CHP	1 (0.1%)
R monotherapy	10 (0.7%)
R-EPOCH with IT vs IV MTX	1 (0.1%)
R-CHOP	23 (1.5%)
Radiation only	35 (2.3%)
Small molecular inhibitor(s) with chemotherapy	17 (1.1%)
Small molecular inhibitor(s) without chemo	26 (1.7%)
Steroids alone	1 (0.1%)
Surgery	3 (0.2%)
Tafasitamab + lenalidomide	24 (1.6%)
Treatment <3 months prior to CD19 CAR-T	36 (2.4%)
Treatment <3 months prior to unspecified CAR-T	11 (0.7%)
Treatment >3 months prior to CD19 CAR-T	4 (0.3%)
Treatment >3 months prior to unspecified CAR-T	2 (0.1%)

**Curative treatment intent**	
Curative intent	984 (65.4%)
No curative intent	481 (32.0%)
Unknown	39 (2.6%)
**Maximum number of lines abstracted**	
2	584 (38.8%)
3	404 (26.9%)
4	229 (15.2%)
5	154 (10.2%)
6	71 (4.7%)
7	33 (2.2%)
8	16 (1.1%)
9	7 (0.5%)
10	3 (0.2%)
11	1 (0.1%)
14	1 (0.1%)
18	1 (0.1%)
Mean (SD)	3.316 (1.559)
Median (IQR)	3.000 (2, 4)
**Treatment location**	
Primary LEO Center	1123 (74.9%)
Other Academic Medical Center	131 (8.7%)
Health System Affiliated Medical Center	94 (6.3%)
Other Community Medical Center	136 (9.1%)
Unknown	16 (1.1%)
Missing data	4

Abbreviations: BTK, Bruton's tyrosine kinase; CAR-T, chimeric antigen receptor T-cell therapy; CEOP, cyclophosphamide, etoposide, vincristine, and prednisone; CEPP, cyclophosphamide, etoposide, procarbazine, and prednisone; CHP, cyclophosphamide, doxorubicin, and prednisone; CHOP, cyclophosphamide, doxorubicin, vincristine, and prednisone; CNS, central nervous system; DHAP, dexamethasone, high-dose cytarabine and cisplatin; DLBCL, diffuse large B-cell lymphoma; EBV, Epstein-Barr virus; ESHAP, etoposide, methylprednisolone, high-dose cytarabine and cisplatin; EPOCH, etoposide, prednisone, vincristine, cyclophosphamide, and doxorubicin; FISH, fluorescent in situ hybridization; gem-ox, gemcitabine and oxaliplatin; GDP, gemcitabine, dexamethasone and platinum agent; GN, gemcitabine and vinorelbine; ICE, ifosfamide, carboplatin, and etoposide; ImID, immunomodulatory drug; IT, intrathecal; IV, intravenous; LEO, Lymphoma Epidemiology of Outcomes; MER, Mayo Epidemiology Resource; NOS, not otherwise specified; PET, positron emission tomography; pola, polatuzumab vedotin; R-, rituximab; RUCC, Rural-Urban Continuum Codes; SD, standard deviation; WHO, World Health Organization.

Supplemental Table 4. Patient characteristics at second-line therapy (2L) for large B-cell lymphoma (LBCL) by Treatment Era

	2002-2010 (N=275)	2011-2017 (N=571)	2018-2022 (N=658)
Demographics			
**LEO Cohort study site**			
Cornell University	1 (0.4%)	27 (4.7%)	62 (9.4%)
Emory University	0 (0.0%)	22 (3.9%)	92 (14.0%)
University of Iowa	57 (20.7%)	41 (7.2%)	44 (6.7%)
Mayo Clinic	196 (71.3%)	187 (32.7%)	44 (6.7%)
MD Anderson	2 (0.7%)	113 (19.8%)	182 (27.7%)
University of Miami	0 (0.0%)	10 (1.8%)	69 (10.5%)
University of Rochester Medical Center	0 (0.0%)	25 (4.4%)	53 (8.1%)
Washington University	19 (6.9%)	146 (25.6%)	112 (17.0%)
**Race**			
White	255 (92.7%)	489 (85.6%)	536 (81.5%)
Black or African American	5 (1.8%)	33 (5.8%)	54 (8.2%)
Asian	1 (0.4%)	13 (2.3%)	29 (4.4%)
American Indian or Alaskan Native	0 (0.0%)	1 (0.2%)	1 (0.2%)
Other	1 (0.4%)	17 (3.0%)	15 (2.3%)
Unknown/Missing	13 (4.7%)	18 (3.2%)	23 (3.5%)
**Ethnicity**			
Non-Hispanic	182 (66.2%)	480 (84.1%)	558 (84.8%)
Hispanic or Latinx	2 (0.7%)	30 (5.3%)	82 (12.5%)
Unknown/Missing	91 (33.1%)	61 (10.7%)	18 (2.7%)
**Gender**			
Female	91 (33.1%)	210 (36.8%)	230 (35.0%)
Male	184 (66.9%)	361 (63.2%)	428 (65.0%)
**Age at diagnosis (years)**			
Mean (SD)	59.5 (13.6)	59.3 (12.6)	62.1 (12.9)
Median (IQR)	62 (52, 69)	60 (52, 68)	64 (55, 71)
Range	19 - 89	18 - 90	19 - 91
18-59	108 (39.3%)	235 (41.2%)	206 (31.3%)
60-69	96 (34.9%)	179 (31.3%)	205 (31.2%)
70-79	58 (21.1%)	128 (22.4%)	194 (29.5%)
80+	13 (4.7%)	29 (5.1%)	53 (8.1%)
**RUCC 2013 status**			
Metropolitan	157 (57.5%)	381 (69.3%)	521 (82.2%)
Non-metropolitan	116 (42.5%)	169 (30.7%)	113 (17.8%)
Missing data	2	21	24
Disease Characteristics			
**Stage**			
I or II	57 (22.0%)	101 (19.6%)	116 (19.2%)
III or IV	202 (78.0%)	415 (80.4%)	487 (80.8%)
Missing data	16	55	55
**ECOG performance status**			

0-1	173 (80.5%)	407 (85.1%)	487 (83.0%)
2-4	42 (19.5%)	71 (14.9%)	100 (17.0%)
Missing data	60	93	71
**Serum lactate dehydrogenase**			
Not Elevated	72 (42.1%)	245 (53.0%)	220 (40.2%)
Elevated	99 (57.9%)	217 (47.0%)	327 (59.8%)
Missing data	104	109	111
**B symptoms**			
None Reported	259 (94.2%)	527 (92.3%)	613 (93.2%)
Yes	16 (5.8%)	44 (7.7%)	45 (6.8%)
**Extranodal sites**			
0-1	194 (72.9%)	398 (72.6%)	422 (66.7%)
2+	72 (27.1%)	150 (27.4%)	211 (33.3%)
Missing data	9	23	25
**IPI score**			
0-1	31 (17.5%)	115 (26.2%)	96 (17.9%)
2	49 (27.7%)	128 (29.2%)	134 (25.0%)
3-5	97 (54.8%)	196 (44.6%)	305 (57.0%)
Missing data	98	132	123
**Bone marrow involvement**			
No	121 (44.0%)	346 (60.6%)	394 (59.9%)
Yes	31 (11.3%)	47 (8.2%)	66 (10.0%)
Missing or indeterminate	123 (44.7%)	178 (31.2%)	198 (30.1%)
**Subtype**[Δ]			
DLBCL	248 (90.2%)	463 (81.1%)	507 (77.1%)
HGBL	14 (5.1%)	39 (6.8%)	40 (6.1%)
Double- or triple-hit	13 (4.7%)	69 (12.1%)	111 (16.9%)
**Cell of origin by Hans algorithm**			
GCB	99 (61.1%)	256 (54.7%)	301 (50.9%)
Non-GCB	63 (38.9%)	212 (45.3%)	290 (49.1%)
N-Miss	113	103	67
**FISH rearrangement**			
No double-hit	48 (81.4%)	324 (85.0%)	486 (84.1%)
Double-hit: rearrangements of MYC and BCL2	3 (5.1%)	19 (5.0%)	24 (4.2%)
Triple-hit: rearrangements of MYC, BCL2 and BCL6	8 (13.6%)	38 (10.0%)	68 (11.8%)
Missing data	216	190	80
**Double expression of MYC and BCL2**			
No	60 (85.7%)	156 (55.7%)	204 (39.9%)
Double expressor	10 (14.3%)	124 (44.3%)	307 (60.1%)
Missing data	205	291	147
**Relapsed/refractory status**			
Achieved CR to 1L, relapsed <12 months	50 (18.2%)	103 (18.0%)	129 (19.6%)
Achieved CR to 1L, relapsed ≥12 months	57 (20.7%)	152 (26.6%)	190 (28.9%)
Did not achieve CR to 1L	168 (61.1%)	316 (55.3%)	339 (51.5%)
Treatment Characteristics			

**Prior anthracycline status**			
Yes	253 (92.0%)	550 (96.3%)	633 (96.2%)
No	22 (8.0%)	21 (3.7%)	25 (3.8%)
**Maximum treatment lines abstracted**			
Mean (SD)	3.4 (1.8)	3.4 (1.6)	3.3 (1.4)
Median (IQR)	3 (2, 4)	3 (2, 4)	3 (2, 4)
Range	2 - 18	2 - 14	2 - 9
**Treatment location**			
Primary LEO Center	194 (70.8%)	431 (75.5%)	498 (76.0%)
Other Academic Medical Center	28 (10.2%)	50 (8.8%)	53 (8.1%)
Health System Affiliated Medical Center	7 (2.6%)	22 (3.9%)	65 (9.9%)
Other Community Medical Center	45 (16.4%)	57 (10.0%)	34 (5.2%)
Unknown	0 (0.0%)	11 (1.9%)	5 (0.8%)
Missing data	1	0	3
**Time from diagnosis to 2L (months)**			
Mean (SD)	11.6 (10.9)	17.9 (24.0)	19.3 (27.5)
Median (IQR)	7.7 (4.9, 14.4)	8.3 (5.4, 19.5)	9.7 (6.0, 19.8)
Range	0.9 - 62.7	0.1 - 151.5	0.3 - 228.8
**Treatment at 2L**			
Salvage chemotherapy	191 (69.5%)	398 (69.7%)	373 (56.7%)
CNS-directed regimen	35 (12.7%)	64 (11.2%)	50 (7.6%)
Immune effector cell therapy	0 (0.0%)	4 (0.7%)	91 (13.8%)
Novel therapy	12 (4.4%)	39 (6.8%)	43 (6.5%)
Frontline-like regimen	20 (7.3%)	36 (6.3%)	28 (4.3%)
Minimal therapy	17 (6.2%)	17 (3.0%)	17 (2.6%)
ImID-containing regimen without chemotherapy	0 (0.0%)	13 (2.3%)	36 (5.5%)
Polatuzumab vedotin +/- CD20 +/- bendamustine	0 (0.0%)	0 (0.0%)	20 (3.0%)
**Stem cell transplant (SCT) at 2L**			
Autologous	96 (34.9%)	208 (36.4%)	167 (25.4%)
Allogeneic	5 (1.8%)	12 (2.1%)	4 (0.6%)
None	174 (63.3%)	351 (61.5%)	487 (74.0%)
**Autologous SCT at any treatment line**			
Yes	130 (47.3%)	262 (45.9%)	189 (28.7%)
No	145 (52.7%)	309 (54.1%)	469 (71.3%)
**CAR-T at any treatment line**			
Yes	1 (0.4%)	79 (13.8%)	314 (47.7%)
Prior autologous SCT	*0 (0.0%)*	*2 (0.4%)*	*5 (0.8%)*
No	274 (99.6%)	492 (86.2%)	344 (52.3%)
**Clinical trial**			
Treated off clinical trial	248 (90.8%)	490 (87.3%)	542 (82.9%)
Treated on clinical trial	25 (9.2%)	71 (12.7%)	112 (17.1%)
Missing data	2	10	4

∆ Best available subtype based on WHO at the time of diagnosis, augmented by FISH testing (clinical and research) where available. **Abbreviations**: 1L, first-line; CAR-T, chimeric antigen receptor T-cell therapy; CNS, central nervous system; CR, complete response; DLBCL, diffuse large B-cell lymphoma; ECOG, Eastern Cooperative Oncology Group; GCB, germinal center B cell-like; HGBL, high-grade B-cell lymphoma; ImID, immunomodulatory drug; IPI, International

Prognostic Index; IQR, interquartile range; LEO, Lymphoma Epidemiology of Outcomes; RUCC, Rural-Urban Continuum Codes; SD, standard deviation.

Supplemental Table 5. Patient characteristics at third-line (3L) treatment.

	Overall (N=893)
**Data Access Group**	
Cornell University	54 (6.0%)
Emory University	64 (7.2%)
University of Iowa	90 (10.1%)
Mayo Clinic	242 (27.1%)
MD Anderson	207 (23.2%)
University of Miami	37 (4.1%)
University of Rochester Medical Center	44 (4.9%)
Washington University	155 (17.4%)
**Race**	
White	756 (84.7%)
Black or African American	52 (5.8%)
Asian	30 (3.4%)
American Indian or Alaskan Native	2 (0.2%)
Other	23 (2.6%)
Unknown/Missing	30 (3.4%)
**Ethnicity**	
Non-Hispanic	723 (81.0%)
Hispanic/Latino	67 (7.5%)
Unknown/Missing	103 (11.5%)
**Gender**	
Female	312 (34.9%)
Male	581 (65.1%)
**Age at Diagnosis**	
Mean (SD)	60.445 (13.036)
Median (Q1, Q3)	62.000 (53.000, 70.000)
Range	19.000 - 90.000
**Age Group**	
18-59	311 (34.8%)
60-69	297 (33.3%)
70-79	226 (25.3%)
80+	59 (6.6%)
**RUCC 2013 description**	
Missing	32
Metro	627 (72.8%)
Nonmetro	234 (27.2%)
**Stage**	
Missing	98
I/II	153 (19.2%)
III/IV	642 (80.8%)

**ECOG PS**	
Missing	129
0-1	590 (77.2%)
2+	174 (22.8%)
**Serum lactate dehydrogenase**	
Missing	151
Not elevated	274 (36.9%)
Elevated	468 (63.1%)
**B symptoms**	
None reported	850 (95.2%)
Yes	43 (4.8%)
**Extranodal sites**	
Missing	38
0-1	573 (67.0%)
2+	282 (33.0%)
**IPI**	
Missing	204
0-1	120 (17.4%)
2	155 (22.5%)
3-5	414 (60.1%)
**Bone Morrow Involvement**	
Missing	13
Yes, DLBCL present per path	35 (4.0%)
Yes, indolent/low-grade B-cell only	3 (0.3%)
No (per path confirmation)	168 (19.1%)
Pathology indeterminate	7 (0.8%)
PET/ radiographic positive, no biopsy performed	27 (3.1%)
No biopsy/ unable to obtain records	353 (40.1%)
PET/radiographic negative, no biopsy performed	284 (32.3%)
Yes, other	3 (0.3%)
**Bone Marrow Involvement**	
No	452 (50.6%)
Yes	68 (7.6%)
Missing/Indeterminate	373 (41.8%)
**Subtype**$^{\Delta}$	
DLBCL	745 (83.4%)
DLBCL + FL	4 (0.4%)
DLBCL + Other	1 (0.1%)
HGBL	143 (16.0%)
**Cell of origin (by Hans algorithm)**	
Missing	590
GCB	80 (26.4%)

Non-GCB	86 (28.4%)
Missing/Unknown	134 (44.2%)
Not Applicable	3 (1.0%)
**FISH+ for MYC and BCL2 and/or BCL6**	
N-Miss	282
No	483 (79.1%)
Double- / triple-hit	128 (20.9%)
**FISH+ for MYC and BCL2**	
N-Miss	280
No	509 (83.0%)
Double-hit	104 (17.0%)
**Double expression of MYC and BCL2**	
Missing	343
No	237 (43.1%)
Double-expressor	313 (56.9%)
**Primary Refractory Status to 1L**	
CR, Relapsed < 12 months	173 (19.4%)
CR, Relapsed >= 12 months	192 (21.5%)
not CR	528 (59.1%)
**Prior anthracycline status**	
No	31 (3.5%)
Yes	862 (96.5%)
**Overall survival (years)**	
N	893
Events	633
Median survival	0.75
Median follow-Up	3.184
**Max number of lines abstracted**	
Mean (SD)	4.162 (1.474)
Median (Q1, Q3)	4.000 (3.000, 5.000)
Range	3.000 - 18.000
**Max number of lines abstracted**	
3	386 (43.2%)
4	224 (25.1%)
5	153 (17.1%)
6	71 (8.0%)
7	31 (3.5%)
8	16 (1.8%)
9	6 (0.7%)
10	3 (0.3%)
11	1 (0.1%)
14	1 (0.1%)

18	1 (0.1%)
**Treatment era for 3L**	
2002-2010	139 (15.6%)
2011-2017	307 (34.4%)
2018-2022	447 (50.1%)
**Treatment Location**	
Missing	5
Primary LEO center	714 (80.4%)
Other academic medical center	69 (7.8%)
Health system-affiliated medical center	31 (3.5%)
Other community medical center	66 (7.4%)
Unknown	8 (0.9%)
**Time from diagnosis to 3L (months)**	
Mean (SD)	21.772 (25.900)
Median (IQR)	12.122 (8.180, 22.996)
Range	2.004 - 188.995
**Treatment Group**	
Anthracycline-containing regimen without CD20	1 (0.1%)
Bispecific antibody	6 (0.7%)
CNS-directed	45 (5.0%)
EPOCH +/- CD20	7 (0.8%)
ImID-based regimen without chemotherapy	53 (5.9%)
Immune effector cell	208 (23.3%)
Intensive	26 (2.9%)
LD chemo only without CAR-T	1 (0.1%)
LD chemo without CAR-T, with bridging	2 (0.2%)
Loncastuximab tesirine	12 (1.3%)
Minimal therapy	108 (12.1%)
Non-anthracycline-containing chemotherapy +/- CD20	72 (8.1%)
Novel therapeutic(s) with or without chemotherapy	96 (10.8%)
Polatuzumab vedotin +/- CD20 +/- bendamustine	38 (4.3%)
R-CHOP	5 (0.6%)
Salvage chemotherapy +/- CD20	212 (23.7%)
Selinexor	1 (0.1%)
**Stem cell transplant (SCT)**	
Allogeneic	5 (0.6%)
Autologous	68 (7.6%)
None	820 (91.8%)
**Ever received autologous SCT**	
No	628 (70.3%)
Yes	265 (29.7%)
**Ever received CAR-T**	

No	549 (61.5%)
Yes	344 (38.5%)
Prior autologous SCT	77 (8.6%)
**Clinical Trial Status**	
Missing	16
Treated off clinical trial	716 (81.6%)
Treated on clinical trial	161 (18.4%)

∆ Best available subtype based on WHO at the time of diagnosis, augmented by FISH testing (clinical and research) where available.

Abbreviations: 1L, first-line; CAR-T, chimeric antigen receptor T-cell therapy; CNS, central nervous system; CR, complete response; DLBCL, diffuse large B-cell lymphoma; ECOG PS, Eastern Cooperative Oncology Group performance status; EPOCH, etoposide, prednisone, vincristine, cyclophosphamide, and doxorubicin; FISH, fluorescent in situ hybridization; FL, follicular lymphoma; GCB, germinal center B cell-like; HGBL, high-grade B-cell lymphoma; IPI, International Prognostic Index; ImID, immunomodulatory drug; IQR, interquartile range; LD, lymphodepleting; LEO, Lymphoma Epidemiology of Outcomes; PET, positron emission tomography; Q, quartile; RUCC, Rural-Urban Continuum Codes; SD, standard deviation.

Supplemental Table 6. Reasons for failure to receive stem cell transplant (SCT) and/or CAR-T in patients with relapsed/refractory LBCL intended for curative therapy.

	2L (N=398)	3L (N=158)	4L+ (N=71)
**Combined SCT/CAR-T planned but not received reason**			
Missing reason	52	25	14
CAR-T manufacturing failure	1 (0.3%)	0 (0.0%)	3 (5.3%)
Inadequate response to / progression on pre-CAR-T (bridging) therapy	4 (1.2%)	22 (16.5%)	12 (21.1%)
Inadequate response to / progression on salvage therapy	231 (66.8%)	67 (50.4%)	21 (36.8%)
Inadequate response to / progression on salvage therapy or CAR-T bridging	66 (19.1%)	28 (21.1%)	16 (28.1%)
SCT collection failure	8 (2.3%)	1 (0.8%)	0 (0.0%)
Treatment complication/ did not meet criteria for CAR-T	4 (1.2%)	6 (4.5%)	3 (5.3%)
Treatment complication/ did not meet criteria for SCT	24 (6.9%)	5 (3.8%)	2 (3.5%)

Abbreviations: CAR-T, chimeric antigen receptor T-cell therapy; LBCL, large B-cell lymphoma; 2L, second-line therapy; 3L, third-line therapy; 4L+, fourth-line or greater therapy

Supplemental Table 7. Characteristics of LBCL patients who underwent autologous stem cell transplant (autoSCT) or CAR-T at third-line therapy (3L).

	AutoSCT (N=68)	CAR-T (N=206)
**Age (years)**		
18-59	38 (55.9%)	70 (34.0%)
60+	30 (44.1%)	136 (66.0%)
**Stage**		
Missing	3	24
I/II	16 (24.6%)	37 (20.3%)
III/IV	49 (75.4%)	145 (79.7%)
**ECOG performance status**		
Missing	12	17
0-1	47 (83.9%)	164 (86.8%)
2+	9 (16.1%)	25 (13.2%)
**Serum lactate dehydrogenase**		
Missing	8	17
Not Elevated	24 (40.0%)	77 (40.7%)
Elevated	36 (60.0%)	112 (59.3%)
**B symptoms**		
None reported	61 (89.7%)	197 (95.6%)
Yes	7 (10.3%)	9 (4.4%)
**Extranodal sites**		
Missing	1	10
0-1	55 (82.1%)	138 (70.4%)
2+	12 (17.9%)	58 (29.6%)
**IPI**		
Missing	15	35
0-1	19 (35.8%)	36 (21.1%)
2	14 (26.4%)	43 (25.1%)
3-5	20 (37.7%	92 (53.8%)
**3L treatment prior to auto SCT**		
CNS-directed	6 (8.8%)	-
EPOCH +/- CD20	1 (1.5%)	-
ImID-based regimen without chemotherapy	1 (1.5%)	-
Immune effector cell	0 (0.0%)	-
Intensive	10 (14.7%)	-
Minimal therapy	4 (5.9%)	-
Non-anthracycline-containing chemotherapy +/- CD20	4 (5.9%)	-
Novel therapeutic(s) with or without chemotherapy	5 (7.4%)	-
Pola +/- CD20 +/- bendamustine	1 (1.5%)	-
Salvage chemotherapy +/- CD20	36 (52.9%)	-

Clinical trial status		
Missing	0	2
Treated off clinical trial	57 (83.8%)	154 (75.5%)
Treated on clinical trial	11 (16.2%)	50 (24.5%)

Abbreviations: LBCL, large B-cell lymphoma; CAR-T, chimeric antigen receptor T-cell therapy; ECOG, Eastern Cooperative Oncology Group; IPI, International Prognostic Index; CNS, central nervous system; EPOCH, etoposide, prednisone, vincristine, cyclophosphamide, and doxorubicin; ImID, immunomodulatory drug; pola, polatuzumab vedotin

Supplemental Table 8. Survival outcomes in subsets of interest at second-line therapy (2L).

Variable	Stratum	Outcome	N (%)	Median survival (months)	HR
2L Treatment Group	*Salvage therapy*	*OS*	*962 (64.0%)*	*18.4 (16.72,25.46)*	*ref*
	CNS-directed	OS	149 (9.9%)	10.38 (6.8,NA)	0.95 (0.76, 1.19)
	Frontline-like regimes	OS	84 (5.6%)	9.13 (6.47,19.28)	1.09 (0.83, 1.44)
	ImID-containing regimen without chemo	OS	49 (3.3%)	18.92 (11.04,83.28)	1.66 (1.15, 2.38)
	Immune effector cell	OS	95 (6.3%)	17.38 (13.11,40.34)	0.94 (0.70, 1.26)
	Minimal therapy	OS	51 (3.4%)	18.99 (9.07,24.8)	0.98 (0.68, 1.41)
	Novel Therapy	OS	94 (6.2%)	19.58 (15.8,NA)	1.27 (0.98, 1.65)
	Pola +/- CD20 +/- bendamustine	OS	20 (1.3%)	26.48 (14.91,66.43)	1.99 (1.12, 3.53)
2L clinical trial status	*Off clinical trial*	*OS*	*1280 (86.0%)*	*28.68 (19.58,66.29)*	*ref*
	On clinical trial	OS	208 (14.0%)	17.38 (16.13,21.06)	0.81 (0.67, 0.98)
Treatment Era	*2002-2010*	*OS*	*275 (18.3%)*	*12.32 (9.43,15.57)*	*ref*
	2011-2017	OS	571 (38.0%)	20.76 (16.56,27.79)	0.78 (0.66, 0.92)
	2018-2022	OS	658 (43.8%)	20.8 (17.38,30.91)	0.71 (0.59, 0.84)
Gender/Sex	*female*	*OS*	*531 (35.3%)*	*18.99 (16.36,27.79)*	*ref*
	male	OS	973 (64.7%)	17.77 (16.43,22.67)	1.03 (0.90, 1.18)
Age Group	*18-59*	*OS*	*549 (36.5%)*	*11.53 (8.28,15.54)*	*ref*
	60-69	OS	480 (31.9%)	15.93 (13.27,22.77)	1.09 (0.93, 1.28)
	70-79	OS	380 (25.3%)	22.67 (17.87,30.09)	1.33 (1.13, 1.58)
	80+	OS	95 (6.3%)	21.16 (17.02,32.36)	2.01 (1.56, 2.61)
Race	*White*	*OS*	*1280 (85.1%)*	*12.78 (12.78,NA)*	*ref*
	Black or African American	OS	92 (6.1%)	21.85 (9.43,NA)	0.80 (0.60, 1.08)
	Asian	OS	43 (2.9%)	17.35 (8.87,NA)	0.94 (0.62, 1.43)
	American Indian or Alaskan Native	OS	2 (0.1%)	18.3 (9.13,67.87)	0.68 (0.10, 4.82)
	Other	OS	33 (2.2%)	26.08 (17.08,NA)	0.86 (0.54, 1.38)
	Unknown/Missing	OS	54 (3.6%)	17.87 (16.46,21.65)	0.98 (0.69, 1.39)
Stage	*I/II*	*OS*	*274 (19.9%)*	*34.3 (26.08,59.33)*	*ref*
	III/IV	OS	1104 (80.1%)	17.05 (15.41,19.28)	1.39 (1.16, 1.66)

ECOG PS	*0-1*	*OS*	*1067 (83.4%)*	*7.16 (6.34,9)*	*ref*
	2-4	OS	213 (16.6%)	26.08 (20.93,32.39)	2.16 (1.82, 2.57)
LDH	*Not Elevated*	*OS*	*537 (45.5%)*	*54.14 (34.3,66.43)*	*ref*
	Elevated	OS	643 (54.5%)	11.3 (10.12,13.67)	1.87 (1.60, 2.18)
Extranodal Sites	*0-1*	*OS*	*1014 (70.1%)*	*15.11 (11.53,17.35)*	*ref*
	2+	OS	433 (29.9%)	22.14 (17.77,28.32)	1.24 (1.08, 1.43)
IPI	*0-1*	*OS*	*242 (18.5%)*	*85.91 (51.64,NA)*	*ref*
	2	OS	311 (27.0%)	48.72 (26.91,73.65)	1.29 (1.01,1.65)
	3-5	OS	598 (52.0%)	10.51 (9.13,13.07)	2.54 (2.05, 3.15)
Subtype[Δ]	*DLBCL*	*OS*	*1218 (81.0%)*	*11.17 (9.66-13.47)*	*ref*
	DH/TH	OS	193 (12.8%)	13.07 (8.02-26.91)	1.58 (1.31, 1.91)
	HBGL	OS	93 (6.2%)	21.85 (18.33-26.61)	1.33 (1.03, 1.72)
Hans COO	*GCB*	*OS*	*656 (53.7%)*	*22.73 (17.58,32.39)*	*ref*
	Non-GCB	OS	565 (46.3%)	17.18 (15.37,22.14)	0.92 (0.79, 1.06)
Primary R/R status to 1L	*CR, Relapsed >= 12 months*	*OS*	*399 (26.5%)*	*53.42 (48.72,75.76)*	*ref*
	CR, Relapsed < 12 months	OS	282 (18.8%)	21.06 (17.05,34.17)	1.41 (1.14, 1.74)
	not CR	OS	823 (54.7%)	11.76 (10.41,13.5)	2.06 (1.74, 2.43)
Diagnosis to 2L treatment interval	*0-36*	*OS*	*1328 (88.3%)*	*51.64 (36.24,83.61)*	*ref*
	36+	OS	176 (11.7%)	16.66 (15.34,18.56)	0.63 (0.50, 0.78)
2L Treatment Group	*Salvage Therapy*	*EFS*	*962 (64.0%)*	*4.3 (3.61,5.19)*	*ref*
	CNS directed	EFS	149 (9.9%)	2.07 (1.38,8.44)	0.75 (0.61, 0.92)
	Frontline-like regimes	EFS	84 (5.6%)	3.84 (2.6,5.03)	1.15 (0.90, 1.46)
	ImID-containing regimen without chemo	EFS	49 (3.3%)	2.83 (2.33,4.14)	1.28 (0.94, 1.75)
	Immune effector cell	EFS	95 (6.3%)	3.4 (2.83,5.22)	0.68 (0.52, 0.88)
	Minimal therapy	EFS	51 (3.4%)	3.88 (2.99,5.55)	1.54 (1.16, 2.05)
	Novel Therapy	EFS	94 (6.2%)	9.33 (6.44,15.11)	1.17 (0.93, 1.47)
	Pola +/- CD20 +/- bendamustine	EFS	20 (1.3%)	5.65 (3.91,13.63)	1.76 (1.07, 2.88)
2L Clinical trial Status	*Off Clinical Trial*	*EFS*	*1280 (86.0%)*	*7.36 (5.26,10.12)*	*ref*
	On Clinical Trial	EFS	208 (14.0%)	4.14 (3.65,4.63)	0.74 (0.62, 0.88)
Treatment Era	*2002-2010*	*EFS*	*275 (18.3%)*	*3.88 (3.06,5.06)*	*ref*

	2011-2017	EFS	571 (38.0%)	5.22 (4.14,6.21)	0.84 (0.72, 0.99)
	2018-2022	EFS	658 (43.8%)	4.14 (3.55,4.83)	0.97 (0.83, 1.14)
Gender/Sex	*female*	*EFS*	*531 (35.3%)*	*4.37 (3.61,5.22)*	*ref*
	male	EFS	973 (64.7%)	4.3 (3.84,5.09)	1.01 (0.90, 1.14)
Age Group	*18-59*	*EFS*	*549 (36.5%)*	*3.19 (2.27,4.37)*	*ref*
	60-69	EFS	480 (31.9%)	4.04 (3.19,4.96)	1.05 (0.91, 1.20)
	70-79	EFS	380 (25.3%)	4.89 (4.17,5.88)	1.17 (1.01, 1.36)
	80+	EFS	95 (6.3%)	4.63 (3.61,5.65)	1.57 (1.24, 1.98)
Race	*White*	*EFS*	*1280 (85.1%)*	*1.56 (1.45,NA)*	*ref*
	Black or African American	EFS	92 (6.1%)	3.81 (2.5,12.65)	0.78 (0.61, 1.01)
	Asian	EFS	43 (2.9%)	4.3 (2.66,8.94)	1.09 (0.78, 1.52)
	American Indian or Alaskan Native	EFS	2 (0.1%)	4.89 (3.75,10.02)	3.40 (0.85, 13.65)
	Other	EFS	33 (2.2%)	5.81 (4.01,8.34)	1.07 (0.73, 1.56)
	Unknown/Missing	EFS	54 (3.6%)	4.2 (3.84,4.96)	0.96 (0.71, 1.29)
Stage	*I/II*	*EFS*	*274 (19.9%)*	*4.66 (3.84,6.27)*	*ref*
	III/IV	EFS	1104 (80.1%)	4.47 (3.94,5.19)	1.09 (0.93, 1.26)
ECOG PS	*0-1*	*EFS*	*1067 (83.4%)*	*2.6 (2.04,3.02)*	*ref*
	0-2	EFS	213 (16.6%)	5.62 (4.99,6.44)	1.75 (1.49, 2.05)
LDH	*Not Elevated*	*EFS*	*537 (45.5%)*	*8.28 (6.9,11.14)*	*ref*
	Elevated	EFS	643 (54.5%)	3.19 (2.79,3.75)	1.61 (1.41, 1.84)
Extranodal Sites	*0-1*	*EFS*	*1014 (70.1%)*	*3.81 (3.15,4.63)*	*ref*
	2+	EFS	433 (29.9%)	4.83 (4.17,5.72)	1.21 (1.07, 1.37)
IPI	*0-1*	*EFS*	*242 (18.5%)*	*8.57 (5.75,12.32)*	*ref*
	2	EFS	311 (27.0%)	8.15 (6.44-11.07)	1.03 (0.84, 1.26)
	3-5	EFS	598 (52.0%)	3.45 (2.99-3.88)	1.75 (1.47, 2.08)
Subtype^Δ	*DLBCL*	*EFS*	*1218 (81.0%)*	*2.56 (1.74-3.19)*	*ref*
	DH/TH	EFS	193 (12.8%)	2.73 (2.27-5.98)	1.67 (1.41, 1.97)
	HBGL	EFS	93 (6.2%)	5.26 (4.73-5.91)	1.18 (0.93, 1.49)
Hans COO	*GCB*	*EFS*	*656 (53.7%)*	*4.86 (3.94,5.95)*	*ref*
	Non GCB	EFS	565 (46.3%)	4.11 (3.52,4.73)	0.93 (0.81, 1.05)
Primary R/R status to 1L	*CR, Relapsed >= 12 months*	*EFS*	*399 (26.5%)*	*13.21 (11.14,16.85)*	*ref*
	CR, Relapsed < 12 months	EFS	282 (18.8%)	4.85 (3.91,6.8)	1.47 (1.24, 1.76)
	not CR	EFS	823 (54.7%)	2.63 (2.37,2.99)	2.01 (1.74, 2.31)
Diagnosis to 2L treatment interval	*0-36*	*EFS*	*1328 (88.3%)*	*13.44 (8.44,23.85)*	*ref*
	36+	EFS	176 (11.7%)	3.88 (3.45,4.27)	0.66 (0.55, 0.80)

Δ Best available subtype based on WHO at the time of diagnosis, augmented by FISH testing (clinical and research) where available. **Abbreviations:** 1L, first-line therapy; 2L, second-line therapy; CNS, central nervous system; COO, cell of origin; CR, complete response; DH, double-hit; DLBCL, diffuse large B-cell lymphoma; ECOG PS, Eastern Cooperative Oncology Group

performance status; EFS, event-free survival; GCB, germinal center B cell-like; HGBL, high-grade B-cell lymphoma; HR, hazard ratio; ImID, immunomodulatory drug; IPI, International Prognostic Index; LDH, lactate dehydrogenase; OS, overall survival; pola, polatuzumab vedotin; ref, reference; R/R, relapsed/refractory; TH, triple-hit.

Supplemental Methods

Variables abstracted

We abstracted demographic characteristics at diagnosis (age, race/ethnicity, geographic location, self-reported gender); date of diagnosis; clinical characteristics at diagnosis and at initiation of each line of treatment (disease stage, body mass index [BMI] defined as weight [kg] divided by height2 [m], components of IPI, B symptoms, bone marrow involvement, presence of extranodal sites [including CNS], and selected lab values [lactate dehydrogenase (LDH), hemoglobin, platelets, absolute lymphocyte count (ALC), absolute neutrophil count (ANC), absolute monocyte count (AMC), white blood cell count (WBC), aspartate aminotransferase (AST), alanine aminotransferase (ALT), bilirubin, creatine (and creatinine clearance by Cockcroft-Gault); and other comorbidities at diagnosis and at initiation of each line of treatment (see Appendix). Treatment details abstracted at each line included date of initiation of treatment, type(s) of treatment (regimen), location of treatment (LEO center or elsewhere), treatment on clinical trial, intent for ASCT and/or CAR-T (and if intended but not received, reason for not receiving), designation of regimen as bridging or salvage prior to consolidative therapy, duration of treatment, response to treatment (including method of assessment), date of first response, reason for treatment termination (e.g., progression/relapse or treatment ineffective, adverse events or unable to tolerate therapy, patient choice, died on therapy, unknown or unable to determine), and date of progression. Tumor characteristics abstracted at initial diagnosis and each subsequent biopsy included lymphoma histology, translocations and overexpression of MYC, BCL2, and BCL6, and cell-of-origin subtype per Hans immunohistochemistry criteria. Finally, outcome data abstracted included date of death or last known follow-up, and for decedents, cause of death. The LEO Statistical Core performed regular central quality control, edit checks, and generated queries and error reports for follow-up at the LEO Centers.

Outcomes

Event-free survival (EFS) was defined as time from initiation of treatment until progression, initiation of a subsequent line of therapy, or death from any cause. Time to next treatment (TTNT) was defined as time from initiation of treatment until initiation of the next line of therapy. Duration of response (DOR) was defined as the time from date of first response to progression or death from any cause (in patients with response. Responses were assessed by positron emission tomography (PET) scan, computed tomography (CT) scan, and by clinician assessment. These variables were also used to define treatment outcome-related groupings (e.g., primary refractory status, etc).

LEO Main Cohort Description

The LEO cohort study is a prospective observational cohort that has enrolled 12,531 newly diagnosed patients with NHL from July 2015 through December 2024. The LEO cohort expanded an ongoing cohort of 4,856 NHL patients enrolled from September 2002 through June 2015 as the Molecular Epidemiology Resource (MER) in the University of Iowa/Mayo Clinic Lymphoma Specialized Program of Research Excellence (SPORE); the MER was then absorbed into LEO as the LEO-MER subcohort. All participants were contacted every 6 months after diagnosis for the first 3 years, then annually thereafter to ascertain disease progression/relapse, transformation, re-treatment and second cancers. These reports were validated against medical records, and were used to identify eligible patients for this LEO CReWE study. For case identification from LEO institutional databases, two major approaches were utilized: 1) cases who were first diagnosed at the LEO institution in 2002 or later and followed by the center (subsequent clinical course abstracted, including outside records as available); and 2) cases who were first diagnosed in 2002 or later at an outside center and then were first seen at a LEO institution with R/R disease following at least 2 prior lines of systemic therapy. Full details of these data sources are provided in the supplemental methods.

Background. The LEO grant started on 6/23/2015 and opened enrollment into the main LEO cohort on 7/1/2015 at Mayo, Iowa, and Weill Cornell Medicine, 8/1/2015 at MD Anderson and URMC; 9/1/2015 at Emory (including Grady Memorial Hospital); 10/1/2015 at Miami, and 2/1/2016 at Washington University.

Eligibility and enrollment. From 7/1/2015 to 4/30/2020, patients were prospectively recruited at each LEO center by clinicians and study coordinators. Patients with NHL who were newly diagnosed within 6 months

(180 days) of enrollment and age 18 years or older were eligible. All patients approached at each center received a LEO ID after registration into the LEO Clinical Database, with final participation status noted (participated, refused, unable to contact, not eligible); all unique patient identifiers tied to the LEO ID are held locally due to IRB and HIPAA restrictions. Participants signed a “registry” consent which allows future unspecified uses of the tissue, serum, peripheral blood cells, DNA and clinical data for lymphoma and related health research. For this study, we screened all LBCL to identify eligible patients.

Baseline clinical abstraction. LEO coordinators abstracted baseline clinical data, labs, and initial course of therapy. These data were entered into the LEO Clinical Database using Medidata Rave, using a web-based data entry system. Pathology review. A Rave form was generated for each participant’s biopsy (or biopsies), including date, timepoint (diagnostic, relapse), sample type (excisional, core, needle), histology if more than one in the same biopsy, and anatomic location (nodal, extranodal, spleen, etc.). Pathology review was coordinated through the LEO Pathology & Biospecimens Core. All cases were initially reviewed and coded into the WHO Classification for Tumours of Haematopoietic and Lymphoid Tissues by the designated LEO pathologist at each center, and the LEO coordinator entered the data in the Rave Tissue Clinical Abstraction Form. The 2008 WHO Classification (Fourth Edition) was updated in 2016, and this update was incorporated into the LEO review, with diagnoses before 2017 classified according to both the 2008 and 2016 systems. A form is completed for each biopsy of the patient, and up to 3 histopathologies within the same biopsy can be captured, ordered by most involvement of tissue sample to least.

The coordinator completed the Abstracted Subtype Form, the LEO Center Pathology Review Form and the Clinical Pathology Marker Form in Rave, which included: clinical and pathological diagnoses, center pathology review calls and specialty pathology markers. If the LEO Clinical and Pathology diagnoses agree, then a final LEO diagnosis is determined by the center pathologist; if they differ, then the LEO center clinician and pathologist met to assign a final LEO diagnosis.

Enrollment Questionnaire (EQ). All LEO participants complete a self-administered EQ, which is available in English or Spanish and in paper or electronic (Qualtrics) formats. The EQ included the following types of data: Demographic - gender, age, race/ethnicity; Anthropometrics - height, weight, historical weight, recent weight loss. Medical history - selected medical conditions at diagnosis including prior cancers/cancer treatments, cardiovascular and other heart disease, etc.; Patient Reported Outcomes - FACT-G (a validated general quality of life instrument); FACT-LYM (a validated lymphoma-specific quality of life instrument); PROMIS Global Health; Vulnerable Elders Survey (VES)-13; and Scored Patient-Generated Subjective Global Assessment – Short Form (PG-SCA). These data were entered into the Rave database by study coordinators, unless the instrument was completed using electronic data capture (Qualtrics), which automatically uploads the data into Rave.

Follow-up. After enrollment, all participants were contacted every six months (±4 weeks) for the first three years after diagnosis, and then annually thereafter until death or disenrollment. If there is no response after two attempts, participants were contacted by phone (or in clinic) to collect the follow-up data. All follow-up surveys to ascertain current weight, lymphoma status, new therapies, second cancers, onset of new morbidities (heart disease, stroke, diabetes, etc.), falls, hospitalizations, and any changes in physicians. In addition, at the 12-, 24- and 36-month follow-ups, participants also completed the FACT-G and FACT-LYM as well as a financial toxicity survey. Each follow-up contact generated a Patient Reported Follow-Up record in the LEO Rave Database. A majority of deaths were identified as part of active follow-up, and nonresponse triggers search to identify decedents via next of kin, physician, center EHR and tumor registry or (final step) linkage to the National Death Index.

Endpoint Validation. If a patient reports disease progression, recurrence, transformation, new therapy or a second cancer, medical documentation is reviewed locally, and if outside records are required these are requested and reviewed by local LEO study physicians to document the event using a standard protocol. For decedents, clinical documents surrounding death are obtained. For patients who report no change in their disease status and who have not been evaluated at one of our institutions in the last year, we verify this with a short letter to their local medical doctor.

LEO-MER subcohort

Eligibility and enrollment. At both Mayo and Iowa, all consecutive cases of lymphoma who were within 9 months of their initial diagnosis at presentation, a US resident, and age 18 years and older were offered enrollment into the study. All participants provided written informed consent. The IRB protocol was written as a "Registry Consent," which allows future unspecified uses of the tissue, serum, peripheral blood cells, DNA and clinical data for lymphoma and related health research. Enrollment into the MER began on 9/1/2002 at Mayo and on 1/1/2003 at Iowa and closed at both sites on 6/30/2015 (start of LEO Main Cohort), with a total enrollment of 4856 NHLs (total enrollment, including CLL and Hodgkin lymphoma was 6972). For this study, we screened all LBCL to identify eligible patients.

Baseline Clinical Data Abstraction. Clinical data, laboratory values, and initial course of therapy at the time of diagnosis were abstracted from primary medical records on all participants; these variables were based on the NCI's common data elements (CDEs) for lymphoma. Treatments were either entered as individual agents or as a regimen, allowing extraction of specific agents for analysis.

Pathology Review. All cases were reviewed by a hematopathologist and classified based on WHO criteria 20. We collected date of diagnostic biopsy, sampling method (excision, core, etc.), grade, and selected subtype-26 specific pathology variables. Up to 3 WHO subtypes were collected, coded from greatest to least involvement.

Enrollment Questionnaire. All participants completed a self-administered baseline questionnaire, which included race/ethnicity; family history of cancer; history of heart disease, diabetes, hepatitis, shingles, hip fracture, other fracture, osteoporosis, premature menopause, infertility, blood clot, use of blood thinner, organ transplant, and autoimmune disorder. Patient-reported outcomes included the Functional Assessment of Cancer Therapy-General (FACT-G) , the Linear Analogue Self-Assessment (LASA) quality of life assessment, and performance status.

Follow-up. All participants have been systematically contacted every six months (±4 weeks) from the date of original diagnosis for the first three years and then annually thereafter. Follow-up data includes disease recurrence or progression, new treatments, transformation, new cancer diagnoses, and new morbidities; at the 1- and 2-year follow-ups we also obtain data on PROs. All reports of disease recurrence, progression, re-treatment or new cancers were validated against medical records. For decedents, we obtained a copy of the death certificate, as well as medical records immediately preceding death in order to review and assign a cause of death by one of the study physicians using a protocol developed for ECOG.

An active follow-up protocol allows us to maintain regular contact with participants (including changes in home address and physicians), maintain ARMIs (Authorization to Release Medical Information) for patients being followed outside of Mayo or Iowa, efficiently identify and validate new events, and obtain follow-up pathology tissue as needed (e.g., at the time of transformation). We also send an annual newsletter to all participants. However, participants may opt-out of the cohort or be followed only through their physician (no direct contact).

LEO Consortium Institutional Databases

Mayo Clinic. In 1986, the Mayo Lymphoma Group established a clinical database of lymphoma patients seen clinically at Mayo and who had a histologically confirmed diagnosis, either newly diagnosed or in the relapsed/refractory setting. This database includes histology; demographics (age, gender); immunophenotype results; selected laboratory data; and type and dose of chemotherapeutic agents administered to the patient, response and duration of response to therapy; and details on available biospecimens (type, location). The data and follow-up included only what was in the chart. Except for key demographics and histology data, most data items are not completed unless needed for special studies. The database coordinator works closely with the Pathology Department to ensure that all new cases of lymphoma are identified and entered into the database in a timely and accurate manner. All patients in the database were diagnosed and/or treated for NHL at the Mayo Clinic in Rochester. Access to the database for research is granted through a waiver of consent by the IRB, or by an IRB protocol to contact patients for additional information. For this protocol, we screened all r/r

LBCL cases seen in the database from 2010-2022 and not already in the LEO cohort or the LEO/MER subcohort.

University of Iowa. The Lymphoma Innovations ORIEN Network (LION) led by Dr. Link at University of Iowa and Dr. Cohen at Emory is a research project that involves 7 institutions and collaborations with sites that participate in the Oncology Research Information Exchange Network (ORIEN). LION utilizes lymphoma-specific protocols for data and sample collection from patients with FL, DLBCL, and MCL combined with use of data and specimens collected under standard practices of the institutional biorepositories and the Total Cancer Care and LEO protocols. Patients provided written informed consent. We searched the ORIEN database at University of Iowa for eligible LBCL patients. At the University of Iowa, we also conducted a search of the Epic EHR to identify additional patients with LBCL seen at Iowa from 2010-2022 outside of LEO, and then screened these cases and abstracted those with documented 2+ lines of treatment. We obtained a waiver of informed consent for use of this database.

Weill Cornell Medicine. The Weill Cornell Medicine (WCM) Clinical Lymphoma Database, initiated in 2010, is an ongoing, prospective, database of newly diagnosed and r/r cases of lymphoma seen at WCM. The Database was developed in collaboration with investigators at the Mayo Clinic and the University of Iowa and was modeled on the Mayo/Iowa SPORE Molecular Epidemiology Resource (MER). Patients provided written informed consent, and the database is an IRB/HIPAA-compliant, electronic platform that provides secure, web-based access to multi-level users to data that is warehoused on secure servers at WCMC. All data field names, the data dictionary, and the standard operating procedures for the WCM Database were drawn directly from the MER to facilitate data sharing in the future. Like the MER, the WCM Database includes forms for patient consent, demographics, prognostic factors and other lymphoma-related details, medical history, family history, and quality of life and epidemiology surveys. In an ongoing fashion, information regarding lymphoma (treatment, progression, transformation) and medical history, as well as quality of life and outcomes data are collected and stored, including for patients actively followed outside WCM. To identify patients for this study, we screened all r/r LBCL cases in the database from 2010-2015 (LEO cohort started in 2015) and r/r cases new to WCM seen 2010-2022. At WCM , we also conducted a search of the WCM Epic EHR to identify additional patients with LBCL seen at WCM from 2004-2009; we then screened these cases for eligibility for this study. We obtained a waiver of informed consent for use of this database.

Emory. Emory also participated in LION (see above), and we searched the ORIEN database for eligible LBCL patients. To identify eligible cases not in LEO or LION, we searched the Emory Lymphoma Enterprise Architecture Database (LEAD), an open architecture, grid-based data repository that integrates pathology, pharmacy, laboratory, cancer registry, clinical trials, and clinical data from institutional databases. We obtained a waiver of consent for these patients.

University of Miami. At University of Miami we use an IRB protocol to retrospectively perform studies on patients with NHL and HL approved by Miami IRB entitled Clinical and Pathologic Studies in Non-Hodgkin's Lymphoma and Hodgkin Disease (SCCC 2003043). To identify cases not already in LEO for this project, we used two databases: 1) the Florida Cancer registry from which we get a list of patients treated at Sylvester Comprehensive Cancer Center (SCCC) with diagnosis of LBCL. The diagnosis was confirmed by cross-matching these patients with pathology database from which we also get a list of patients with diagnosis of LBCL diagnosed at SCCC and Jackson Memorial Hospital. After cross-matching these databases we went through electronic records to identify patients who received 2 or more different lines of therapy and then to extract the needed data for the proposed study.

MD Anderson. The MD Anderson Lymphoma Outcomes Database (LOD) is a retrospective, observational database of patients with lymphoma who received care at MD Anderson from 2000 to the present. The LOD is stored in a Research Electronic Data Capture database (REDCap) containing records for over 25,000 lymphoma patients. Patient characteristics at diagnosis, disease characteristics, and treatment regimens are available in the database and additional data elements were abstracted from the EHR. For this protocol, we screened all LBCL in the database (2002-present) and not already in the LEO cohort, and abstracted those with documented 2+ lines of treatment. We obtained a waiver of informed consent for this database.

University of Rochester Medical Center (URMC). The University of Rochester Lymphoid Malignancies Database (LMD) is an observational database of patients with lymphoma who received care at the University of Rochester Wilmot Cancer Institute from 2014 to the present. The LMD is stored in a Research Electronic Data Capture database (REDCap) containing records for nearly 5,000 lymphoma patients. Patient characteristics at diagnosis, disease characteristics, and initial treatment regimens were available in the dataset and additional data elements were abstracted from paper-based records and EHRs. For this protocol, we screened all LBCL in the database (2014-present) and not already in the LEO cohort, and abstracted those with documented 2+ lines of treatment. We supplemented this database by searching the EHR as well as adding patients previously enrolled on the NLCS. We obtained a waiver of informed consent for use of this database.

Washington University. The Washington University Lymphoma Banking program was established in 2008 to obtain high-quality, clinically annotated tissue from patients with lymphoid malignancies. Along with collection and storage of cryopreserved tissue samples, clinical and demographic data for over 1200 lymphoma patients is stored in a Research Electronic Data Capture database (REDCap). This database included data on histology; demographics (age, gender, race); initial diagnostic data (stage, ECOG, prognostic indices i.e.) immunophenotype and cytogenetic results; chemotherapeutic/surgical/radiotherapy treatments administered to the patient and the response and duration of response to therapy. For this protocol, we screened all LBCL in the REDCap database not already in LEO cohort, and abstract those with documented 3+ lines of treatment. Patients with a LBCL diagnosis not consented to the Washington University Lymphoma Banking program were identified by interrogating the Command Health EHR database (2006-present). The identified cases were screened for those with documented 2+ lines of treatment and data abstracted. Patient characteristics at diagnosis, disease characteristics, and initial treatment regimens were available in the dataset and additional data elements were abstracted from paper-based records and EHRs.

LEO CReWE Center Enrollment

**Cohort**	**PI(s)**	
LEO Cohort	James Cerhan<br>Christopher Flowers	
**LEO Centers**	**Center PI**	**Number of eligible patients enrolled**
Mayo Clinic	Thomas Habermann	501
MD Anderson	Loretta Nastoupil	612
Washington University	Brad Kahl	251
University of Miami	Izidore Lossos	279
Weill Cornell	Peter Martin	280
University of Iowa	Brian Link	190
Emory University	Jonathon Cohen	313
University of Rochester Medical Center	Jonathan Friedberg	256
